# Barriers and Facilitators for Interprofessional Education in Work-Focused Healthcare: An Integrative Review

**DOI:** 10.1007/s10926-025-10278-3

**Published:** 2025-02-23

**Authors:** Elmi Zwaan, Nina Zipfel, Wietske Kuijer-Siebelink, Shirley Oomens, Sylvia J. van der Burg-Vermeulen

**Affiliations:** 1 Amsterdam UMC, University of Amsterdam, Department of Public and Occupational Health, Amsterdam Public Health Research Institute, Meibergdreef 9, 1105 AZ Amsterdam, the Netherlands; 2 Research on Learning and Education, Radboud University Medical Centre, Radboudumc Health Academy, Nijmegen, the Netherlands; 3 School of Education, HAN University of Applied Sciences, Research Group Responsive Vocational and Professional Education, Nijmegen, the Netherlands; 4 HAN University of Applied Sciences, Occupation and Health Research Group, Nijmegen, the Netherlands

**Keywords:** Work-focused healthcare, Occupational health, Interprofessional education, Interprofessional learning, Integrative review

## Abstract

**Purpose:**

To identify, summarize, and synthesize barriers and facilitators associated with interprofessional education (IPE) for work-focused healthcare professionals such as occupational physicians, social insurance physicians, and labor experts, to inform and stimulate interprofessional collaborative practice within the field of work-focused healthcare.

**Methods:**

An integrative review was conducted to identify studies that report on IPE for work-focused healthcare professionals. Eight databases (APA PsycInfo, CINAHL, Cochrane, Embase, ERIC, Google Scholar, PubMed, Web of Science) were searched until March 2024. Reference lists of included articles were screened. Inclusion criteria were a description of an IPE activity of which at least one group of participants were work-focused healthcare professionals (in training). Barriers and facilitators were structured using the framework of Measurement Instruments for Determinants of Innovation (MIDI).

**Results:**

From 6123 studies, seven were included. Barriers and facilitators were identified for each level of the MIDI framework. For example, at the level of characteristics of the IPE activity, involvement of skilled educators and reflection opportunities facilitated IPE. At the level of characteristics of IPE participants, role misunderstanding and lack of interaction hindered IPE. At the level of the organizational context, connection to work practice facilitated IPE. At the level of socio-political context, lack of support from external organizations hindered IPE.

**Conclusion:**

Insights from this review can inform future IPE development. It is recommended that IPE is integrated in a learning continuum, is connected to daily practice, and includes reflection possibilities and training for interprofessional competencies.

**Supplementary Information:**

The online version contains supplementary material available at 10.1007/s10926-025-10278-3.

## Introduction

Interprofessional collaboration has shown to effectively enhance care and improve patient outcomes (e.g., increased hospital survival, patient well-being, and patient satisfaction) within complex care systems such as primary healthcare [1–3]. Similarly, work-focused healthcare is considered a complex healthcare system [4]. It involves various care providers who are all engaged in tasks such as supporting and guiding sick-listed workers in staying at work and/or returning to work, assessing the work capacity of sick-listed workers, and offering interventions to promote health and well-being at work [5]. Work-focused healthcare is vital for improving work participation and facilitating the return-to-work (RTW) process for individuals who experience absenteeism or prolonged work disability due to illness. This is particularly important as employment and well-being at work are evidently related to perceived health and overall well-being in life [6, 7].

Work-focused healthcare in the context of RTW support goes beyond the medical perspective. Various professionals that focus on work, health, and functioning are involved, such as occupational health physicians, insurance physicians, occupational health nurses, labor experts, and allied health professionals (e.g., rehabilitation counselors, psychologists) [8]. Moreover, other stakeholders that can be involved are, for example, HR professionals, and the employer of the sick-listed worker [8]. All these various stakeholders have distinct roles and perspectives within the work, health, and functioning of sick-listed workers in the RTW process [8]. These differing viewpoints and vested interests can impact the coordination of RTW support [9], potentially leading to a lack of interprofessional collaboration [10]. For example, previous research has shown that (partially) sick-listed workers often perceive a lack of consensus among work-focused healthcare professionals regarding their RTW approach [10]. Workers have expressed a need for better communication between work-focused healthcare professionals and greater consistency in the information provided to them [10]. Therefore, to deliver optimal care and effective RTW support to sick-listed workers, interprofessional collaboration and communication between all involved professionals seems to be essential [9, 11, 12].

In work-focused healthcare, previous literature emphasizes the importance of collaboration between the involved professionals to achieve shared goals in RTW support [13]. However, to date, there are few scientific, evidence-based initiatives aimed at enhancing interprofessional collaboration among work-focused healthcare professionals. One study found that increased multidisciplinary communication between occupational physicians and social insurance physicians contributed to better understanding of each other’s roles [14]. This communication facilitated coordinated work capacity assessments and the development of optimal RTW strategies. As a result, it provided a more effective RTW support for sick-listed workers and, in some cases, led to the modification or prevention of inadequate RTW plans, thereby avoiding unnecessary productivity losses, absenteeism costs and subsequent wage penalties for employers [14].

To enhance interprofessional collaboration and communication within work-focused healthcare, it is essential for professionals in this field to develop key skills, such as relationship building, interaction, and reflective discussion skills [15]. These skills can be acquired through targeted training [16]. Interprofessional education (IPE) has gained widespread support and could serve as a pivotal strategy for equipping work-focused healthcare professionals with essential interprofessional collaboration skills, while also fostering an interprofessional collaborative environment [12, 15]. IPE is defined as the process where individuals from two or more professions learn with, from, and about each other, with the aim of improving interprofessional collaboration and patient/health outcomes [17]. Terms such as interprofessional and multiprofessional are often used interchangeably or incorrectly [18, 19]. However, IPE differs from multiprofessional education in the sense that IPE involves joint setting and evaluation of goals, active interaction among professionals or students from different professions, and the exchange of knowledge and competencies [18, 20]. Multiprofessional, on the other hand, implies a focus on an individual process instead of a collective process [18]. Professionals share information, but work alongside each other and all have separate goals to accomplish [18, 20]. Because of its greater emphasis on collaboration, shared goals, and integrated learning [21], IPE is preferred over multiprofessional education to foster an interprofessional collaborative practice among work-focused healthcare professionals.

Research shows the added value of IPE in primary care. For example, IPE improved attitudes toward, understanding of, and collaboration among general healthcare professionals [22–24]. One study showed that primary care professionals involved in elderly care who participated in an IPE activity reported improved team-skills, and improved attitudes toward and collaboration with other professionals involved in the health or social care of elderly [25]. Evidence supporting the positive effects of IPE on patient outcomes remains limited. A scoping review that synthesized the available literature on this topic identified a positive relationship between IPE and various patient outcomes including length of stay, incidence of medical errors, patient satisfaction, and mortality [26].

However, the application of IPE specifically within the context of work-focused healthcare is a relatively novel area of focus. Most existing research has concentrated on IPE in clinical and primary care settings. While research on team-based approaches is becoming increasingly prevalent in primary healthcare [27], professionals in work-focused healthcare often do not operate in interprofessional teams. In some countries, roles such as providing RTW support and sick leave assessment for certification of sickness benefit are even deliberately separated to avoid conflicts of interest between the RTW support perspective and the social security perspective [28, 29]. Consequently, IPE designed for work-focused healthcare professionals may differ from IPE designed for primary care professionals.

Therefore, this integrative review aimed to provide insights into barriers and facilitators for IPE targeted at work-focused healthcare professionals involved in the RTW process of sick-listed workers, with the goal of informing the future development of IPE activities in this field. The main objective of this integrative literature review was to identify, summarize, and synthesize barriers and facilitators associated with IPE for work-focused healthcare professionals [30]. These insights can inform and stimulate interprofessional education and interprofessional collaborative practice within the field of work-focused healthcare [31].

## Methods

### Study Design

The integrative literature review method was considered the most suitable study design, as it allowed for inclusion of data from a variety of study designs. Moreover, an integrative review aims to gain insights into the current state of research on a given topic [32], synthesize knowledge, assess the applicability of results to practice [33], and recommend future directions for both research and practice [32]. Results are reported in accordance with the Preferred Reporting Items for Systematic Reviews and Meta-Analyses (PRISMA) statement, following them as closely as possible [34] (see Online Resource 1). The protocol for this integrative review has been published in the PROSPERO data base (ID: CRD42022377868).

### Databases and Search Strategy

The following databases were searched for all eligible studies published until March 2024: APA PsycInfo, CINAHL, Cochrane, Embase, ERIC, Google Scholar, PubMed, and Web of Science. No restrictions were placed on publication date, allowing studies from any year to be considered eligible. To ensure a comprehensive search, Google Scholar was considered unsuitable as a single resource [35]. Instead, it is recommended to combine multiple databases and include the first 200 references from Google Scholar [36, 37]. Furthermore, snowball strategies were applied to screen reference lists of included studies against inclusion criteria. Search terms included free-text terms to capture concepts of “interprofessional education” (e.g., ‘interprofessional training’ and ‘cooperative learning’) and “work-focused healthcare” (e.g., ‘occupational health’ and ‘job re-integration’). An information specialist assisted with setting up the search strategy and conducting the search. The search strategy was developed in accordance with the criteria of the Canadian Agency for Drugs and Technologies in Healthcare peer review checklist for search strategies [38]. The search strategy tailored toward each database is available in Online Resource 2.

### Study Selection

Studies eligible for inclusion had to describe the development and/or the implementation of an IPE activity in undergraduate, graduate, workplace learning, or continuing education. An IPE activity could vary in content and duration (e.g., one-hour training, full-semester course, training of multiple days), but at least one group of participants had to consist of work-focused healthcare professionals (in training). Work-focused healthcare encompasses clinical and non-clinical disciplines [39]. Common professionals within work-focused healthcare include occupational physicians, social insurance physicians, labor experts, occupational health nurses, rehabilitation counselors, organizational psychologists, occupational health and safety professionals, and allied health professionals, such as physiotherapists, and primary care psychologists. During the screening stage, the authors also verified whether the IPE activity was interprofessional rather than multiprofessional or multidisciplinary in nature. Studies with a quantitative, qualitative or mixed-methods design were eligible. Only studies written in Dutch, English or German were eligible for inclusion, as these languages aligned with the proficiency of the research team. Not eligible for inclusion were studies describing an IPE activity in which none of the professional groups were work-focused healthcare professionals, studies not describing any IPE or interprofessional training, or studies describing education for only one healthcare professional group.

Deduplication was done in EndNote following the steps suggested by Bramer et al. [40]. The online tool Rayyan [41] was used for screening against inclusion criteria. First, one author (EZ) screened the studies on title. Subsequently, each abstract was screened independently by at least two authors (EZ and SvdB or NZ and SO). Full-texts of the included studies were then screened against inclusion criteria by two authors independently (EZ and NZ). For all screening phases, involved authors discussed the potential eligible studies in case of disagreement, until consensus was reached. If necessary, a third author was consulted to reach consensus.

### Data Extraction and Synthesis

First, data were extracted from studies based on a pre-determined data extraction form. Information such as study characteristics, description of the IPE activity, and participants of the IPE activity was extracted. Second, barriers and facilitators were extracted from included studies. Barriers are factors that hinder successful completion of the IPE activity and reduce the satisfaction of those involved. Facilitators are factors that help to achieve the goals of the IPE activity and contribute to increased participant satisfaction [42]. The data extraction form is available in Online Resource 3. For all studies, data extraction was performed independently by two authors (EZ and NZ). In case of disagreement, the authors discussed the study until consensus was reached. If necessary, a third author (SvdB) was consulted.

Data was synthesized in a narrative form. With this technique, findings from diverse study designs can be categorized into multiple groupings [43]. From tabulated data, groupings were determined, after which data was described in text [44]. The framework of the Measurement Instrument for Determinants of Innovation (MIDI) [45] was used to synthesize data into groupings. This framework contains four broad categories of determinants that can affect implementation of an innovation and, for the purpose of our study, was adapted to the following: characteristics of the IPE activity, characteristics of the participant of the IPE activity, characteristics of the organizational context of the IPE activity, and characteristics of the socio-political context in which the IPE activity takes place (i.e., degree to which the IPE activity fits in with existing regulations and legislations established by authorities).

### Quality Assessment

Quality of studies was assessed by using Hawker’s critical appraisal tool [46]. This tool was deemed most suitable since it can assess methodological quality across different study designs. It contains nine categories to assess methodological quality: abstract and title, introduction and aims, method and data, sampling, data analysis, ethics and bias, results, transferability/generalizability, and implications and usefulness (see Online Resource 4). Overall quality grades were created based on Lorenc et al. [47]. Answers to each of the nine questions (i.e., ‘very poor,’ ‘poor,’ ‘fair,’ ‘good’) were converted to a numerical score by assigning points from 1 (‘very poor’) to 4 (‘good’). For each study, a total score was calculated by adding up all points of the nine questions. Each study could reach a score between 9 and 36 points. The following definitions were used for overall quality grades: low quality (9–24 points), medium quality (24–29 points), high quality (30–36 points) [47]. Studies with low methodological quality were not excluded, since the total amount of included studies is low and the study still described an IPE activity and therefore could contain valuable information regarding barriers and facilitators for IPE. However, results coming from studies with low methodological quality should be interpreted with caution. Quality assessment was done by two authors (EZ and NZ) independently. Disagreements were discussed until consensus was reached.

## Results

**Fig. 1:**
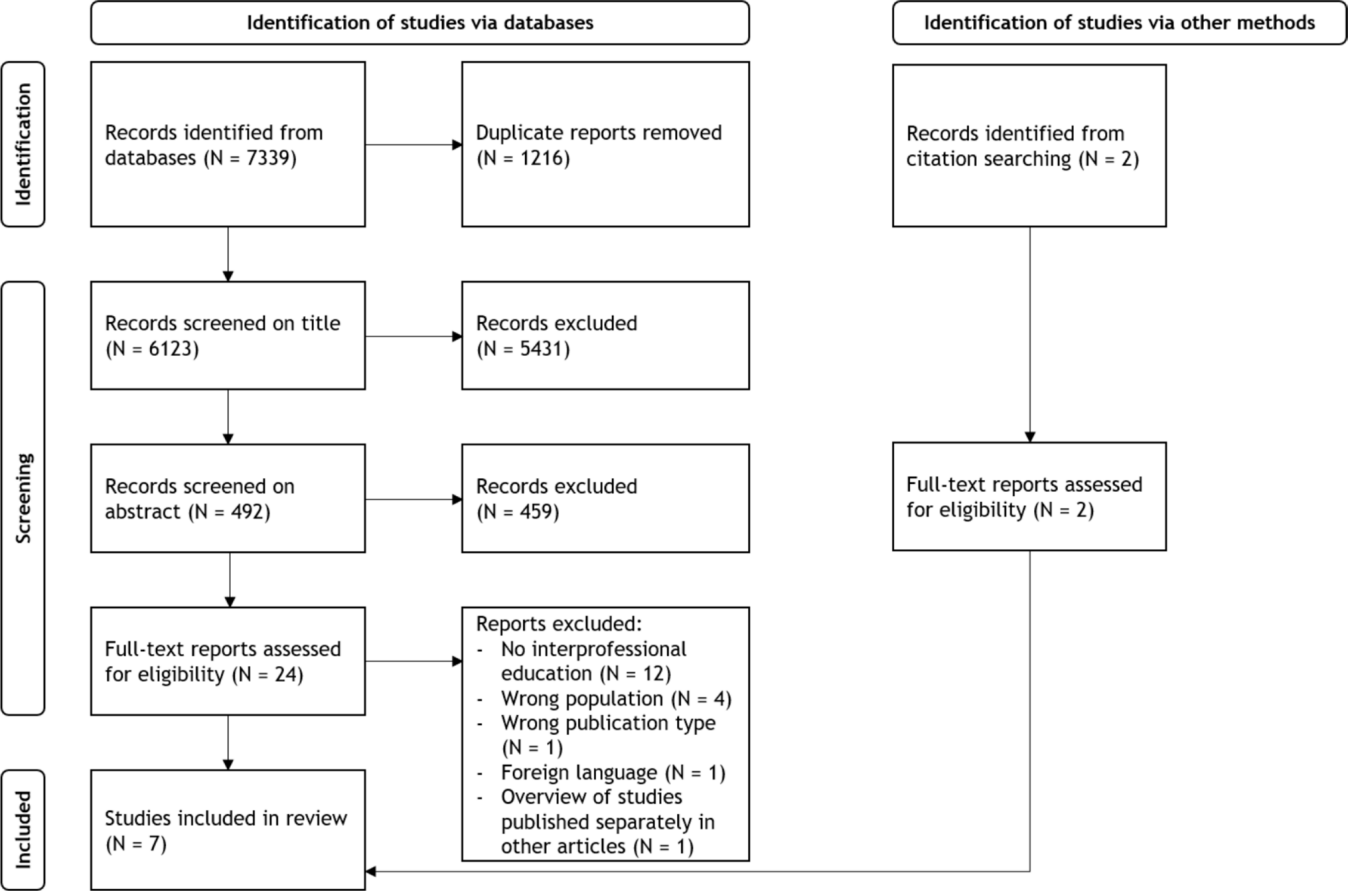
PRISMA flow diagram of study inclusion

Initially, the search yielded N = 7339 eligible studies. Following deduplication, N = 6123 unique studies remained for title and abstract screening. A total of N = 24 studies were screened based on full-text, of which N = 5 were deemed eligible for data synthesis. Citation searching resulted in an additional N = 2 articles eligible for inclusion. Thus, in total, N = 7 studies were included in the data synthesis. For all screening phases, the most common reasons of exclusion were that the educational activity did not qualify as IPE, or the population did not include at least one group of work-focused healthcare professionals. Figure 1 shows the PRISMA flow diagram for inclusion.

### Study characteristics

Study characteristics are presented in Table 1. Study characteristics are presented according to the Interprofessional Learning Continuum (IPLC) Model [48]. This model covers four interrelated components of IPE: the learning continuum, the learning outcomes, health and system outcomes, and enabling or interfering factors [48]. IPE activities of N = 4 studies had the primary aim to increase collaboration between participants [39, 49–51]. One study primarily aimed to train work-focused healthcare professionals from low- and middle-income countries on occupational health in a global context [52]. In this study, the secondary aim was to prepare these professionals for interprofessional collaboration [52]. The IPE activities of remaining studies aimed to present work-focused healthcare students different perspectives of occupational health whereby providing them with the opportunity to learn from each other [53, 54].

**Table 1:** Study characteristics of included papers

References	Author, year, outer setting, inner setting	IPE program aim	Program description, timing	Place on learning continuum Participants, sample size	Learning outcomes	Health and system outcomes
[50]	Böhnke et al., 2005 Germany, Bavarian Academy for Social and Environmental Medicine	Give professionals information and abilities to be applied in their practice regardless of the industry they work in (flexibility, ability to communicate, to collaborate, function socially, ability to work in a team and collaborate with persons from other disciplines)	Three courses. Course A focus on theoretical basis and basics of communication and conflict management; Course B focus on theory collaboration including presentation techniques and group dynamics; Course C focus on interdisciplinary collaboration with role play and cases Six times four hours	Continuing professional development Work-related healthcare professionals: self-employed, at hospitals, other companies, professionals with focus on OHS, professionals working at a court house, professionals working at the institute of unemployment Sample size unknown	*Reaction:* Evaluation showed above average satisfaction for information content, communication, collaboration and communication techniques, interdisciplinary communication, and group dynamics	
[51]	Faber et al., 2005 The Netherlands, intervention vs. control region	Increase collaboration between GPs and OPs in the treatment of patients with low back pain	One main session and two voluntary follow-up sessions. Main session focus on learning to work with a joint collaboration protocol that defines and suggests moments at which collaboration between GP and OP is useful. During the follow-up sessions GPs and OPs practice using the protocol with case studies and discuss daily practice and/or encountered difficulties Main session: four hours Follow-up sessions: two hours each	Continuing professional development Intervention region: 21 GPs, 20 OPs Control region: 28 GPs, 27 OPs	*Collaborative behavior:* No significant differences in amount of contact between OP and GP between intervention and control groups	*Individual health:* No differences in improvement of pain, disability, and quality of life between patients in intervention and control group Control group significantly shorter duration of sick leave compared to intervention group
[54]	Gillespie et al., 2023 USA, Educational Research Center of a university in the Midwest	Explore intersection, practice, influence of culture and diversity of occupational health and safety disciplines. Increase exposure to real world settings	Professional field trip to a field site to which occupational safety and health can be applied (e.g., coal mine, chemical manufacturing plant, laboratory). Reflection on interprofessional field trip experience afterwards Trips varied from 1 to 3 days	Graduate education Students from graduate education programs: industrial hygiene, occupational health nursing, occupational medicine, occupational safety and health engineering, biomonitoring Groups varied from *N* = 5 to *N* = 17	*Reaction:* Students experienced personal value *Attitudes/perceptions:* Reflection on and correction of assumptions about other disciplines *Knowledge/skills:* Students learned about various work environments and policies, and provided input on logistics and planning of trips. Understanding of how successful interprofessional interactions contribute to big picture.. Field trips helped students to meet possible employers and career possibilities	
[39]	McCullagh et al., 2022 USA, University of Michigan	Improve students’ skills in interprofessional collaborative practice	Revised existing multidisciplinary graduate course containing specific, primarily face-to-face, interactive IPE learning activities focused on learning from, with and about each other. Different teaching methods (e.g., case studies, team-based learning, lectures by extramural guest speakers). Interprofessional course faculty. Two group-work assignments in which groups of all participating disciplines were formed Winter semester (approx. 4 months)	Graduate education Graduate students who followed the course in 2019 (*N* = 36): industrial hygiene (*N* = 19), industrial and operations engineering (*N* = 6), occupational health nursing (*n* = 4), occupational epidemiology (*N* = 3), other public health majors (*N* = 2), other engineering majors (*N* = 2) Sample composition and size of 2020 course unknown	*Attitudes/perceptions:* Increase in comfort with working in interprofessional teams Small increase in value of IPE *Knowledge/skills:* Improvement in understanding of roles of participating OHS disciplines	
[49]	Nauta et al., 2006 The Netherlands, Rotterdam, GPs and OPs work places	To realize collaboration between GP and OP in practice	Four day postgraduate program. Day 1: separate activities: knowledge test, own experiences (positive/negative), identify factors impeding contact, cases for homogeneous groups, statements. Day 2: joint activities: presentation of statements for plenary discussion, cases for mixed discussion groups (2 GP, 1 OP), own experiences, making arrangements for working visits. Day 3: working visits: GP visit OP and company, OP visit GP, observation and discussion + practical tasks: work-related health complaints, permission of client, consultation of other discipline, evaluation, report (3 cases). Day 4: conclusions: presentation of task reports, discussion of working visits, general discussion and conclusions, evaluation Four days	Continuing professional development GPs (*N* = 34 at T1, *N* = 33 at T2, *N* = 14 at T3) OPs (*N* = 20 at T1, *N* = 20 at T2, *N* = 10 at T3)	*Attitudes/perceptions:* Short-lasting effect of increased trust of GPs in OPs Increase in understanding of other professional, prejudices not substantiated *Collaborative behavior:* More initiative to contact other professional	
[52]	Radon et al., 2009 Germany, Training centre of the Bavarian Farmer’s Association in Herrsching	Train physicians, nurses, and other healthcare professionals currently working or training in OH in low- and middle-income countries on the most important aspects of OH in the global context	Combination of interactive lectures, case-based learning, readings, presentations by participants, small group sessions, enterprise visits, concept mapping, and hands-on sessions on different topics of OHS in different countries. Lecturers (*N* = 15) were experienced with international audiences and facilitated broad coverage of industrialized and developing countries. Seven tutors assisted the program 90-h program over the course of two weeks, including board and lodging	Continuing professional development Physicians (*N* = 19) Nurses (*N* = 3) Of which *N* = 20 in some way related OH	*Reaction:* IPE activity met expectations. Overall quality of the activity high Satisfaction with content of course, knowledge of presenters, and method of teaching	
[53]	Rosen et al., 2011 USA, New York/New Jersey ERC	Provide students with a multidisciplinary understanding of OSH issues and to give them the opportunity to learn from each other	Industrial tour to several industrial and environmental sites of historical significance. Students get a historical perspective while learning about safety and health issues in those industries. Hazards and environmental issues are identified to demonstrate how important interdisciplinary education is. Major deliverable is participation in development of an interdisciplinary presentation Five to six days, including overnight stays	Foundational education Sample varied over the different tours between nine and 25 undergraduate students, from: occupational medicine, industrial hygiene, occupational safety, ergonomics, public health, and occupational nursing	*Reaction:* Sufficient time to interact with professionals and students from different disciplines during the IPE activity *Knowledge/skills:* IPE activity helped to look outside the box to find solutions for complex problems	

*GP* General practitioner, *OH *Occupational healthcare, *OHS *Occupational health and safety, *OP *Occupational physician

The timing of IPE activities ranged from a one-time, four-hour training with two voluntary follow-up sessions of two hours each [51], to a program spanning several days to two weeks including overnight stays [52–54], to a semester long course [39]. Program activities consisted, for example, of case studies [39, 49–52], small group assignments [39, 52], interactive lectures [39, 52], and work or site visits [49, 52–54].

Regarding position on the learning continuum, the IPE activity of one study was aimed at undergraduate students [53], and two studies were aimed at graduate students [39, 54]. The remaining studies (N = 4) were aimed at continuing professional development [49–52]. Participants in the IPE activities of the included studies comprised of a diverse range of work-focused healthcare professionals, including physicians, nurses, and professionals employed at a national institute for unemployment [50, 52], as well as general practitioners and occupational physicians [49, 51]. Additionally, participants included (under)graduate students from various (work-focused) healthcare disciplines, such as occupational medicine, public health, and industrial hygiene [39, 53, 54].

Studies assessed outcomes at various levels of the IPLC model. All studies assessed outcomes at one or more components of the learning outcomes level: reaction to the IPE activity (e.g., satisfaction with working methods), attitudes and perceptions (e.g., value of interprofessional teams), knowledge and skills (e.g., understanding each other’s role), and collaborative behavior (e.g., frequency of contact). Only one study assessed outcomes at the health and systems outcome level (e.g., severity of back pain, functional disability, and duration of sick leave). Main outcomes for each study can be found in Table 1.

### Quality assessment

Quality scores ranged from 21 (indicating low quality) to 34 (indicating high quality) across the included studies. N = 1 study was rated as low quality [50], N = 3 as medium quality [52–54], and N = 3 as high quality [39, 49, 51]. Results from the study with low methodology should be interpreted with caution. Detailed information regarding the quality assessment can be found in supplemental material 4.

### Facilitators and barriers

Facilitators and barriers for IPE are presented using the MIDI framework of Fleuren et al. [45]. Table 2 presents an overview of facilitators and barriers for each category of the MIDI framework. Facilitators and barriers associated with each category are described below.

**Table 2:** Overview of facilitators and barriers structured according to the MIDI framework

Barrier/facilitator	Theme	Description	References
Characteristics of the IPE activity			
Facilitators	Involvement of skilled and dedicated contributors	Participation and dedication of occupational health professionals, alumni, and external advisory board as lecturer/tutor, and planning/organizing	[39, 54]
		Highly motivated presenters that passed on enthusiasm to participants	[52]
		Experienced faculty ensured that information is conveyed in an understandable manner for all trainees from different disciplines	[53]
		Interaction with specialists from the field helped students connecting with the field and identify employment possibilities	[54]
	Interaction possibilities	Atmosphere where questions are welcomed	[53]
		Possibility to interact with trainees and faculty from different disciplines	[53, 54]
		Beyond-class possibilities for interaction	[53]
	Reflection possibilities	Reporting interprofessional field trip experiences allowed for reflection of learning	[54]
		Structured discussion after the interprofessional activity allowed for reflection on assumptions of other disciplines	[54]
Barriers	Inadequate alignment with previous knowledge and education	Interprofessional field trip should align with students’ own educational program	[54]
	Perceived lack of added value	IPE activity did not contain much new information (already good adherence to guideline)	[51]
Characteristics of the participant of the IPE activity			
Facilitators	Personal qualifications	Social competences of work-focused healthcare professionals. Being mindful of norms and values of professional in IPE activity	[50]
Barriers	Insufficient group interaction skills and commitment	Lacking communication techniques between participants	[50]
		Not optimal group dynamics	[50]
		Language barrier between participants	[52]
	Misunderstanding of the role of other participants	Misunderstanding the role of other participants from different disciplines	[51]
		Experiencing views of participants from other disciplines as inhibitory factor	[51]
Characteristics of the organizational context of the IPE activity			
Facilitators	Practical applicability	IPE activity being a vocational training therefore creating possibility to put learned knowledge into practice	[49]
	Repeated IPE experiences	Possibility to participate in the IPE activity in two consecutive years	[39, 53]
	Hands-on practical experiences and adequate logistics	Hands-on experience (on sites). Helped identification of differences between expectations and reality	[52–54]
		Appropriate work sites in which all disciplines are encountered	[53]
		Desire of visited work sites to participate in the training experience. Welcoming employees at sites	[53, 54]
		Possibility to engage with personnel from sites	[53]
		Work site staff to guide and inform group	[53]
		Extensive planning and an organized itinerary is necessary to identify suitable organizations to visit during an interprofessional field trip	[54]
Barriers	Time-related challenges	Lack of time available for the topic of IPE within the course	[39]
		Course being a one-time program	[49]
	Lack of organizational support	Lack of allocated time and financial support for continuing education	[50]
		Lack of collaboration between university and specialized institutes	[50]
		Financial support only available for specific participants	[52]
	Restrictions due to organizational regulations	Change in course delivery from in person to online due to COVID-19 regulations	[39]
Characteristics of the socio-political context of the IPE activity			
Facilitators	Supported by law	IPE activity is based on a lawful act	[50]
Barriers	Lack of support from external organizations	Restricted access to suitable work sites due to security concerns	[53]

### Facilitators related to the characteristics of the IPE activity

**Involvement of skilled and dedicated educators**. Participation and dedication of occupational health and safety professionals as educators who connected course material to work-focused health practice outcomes, facilitated the success of the IPE activity [39, 52–54]. In one study, professionals working in occupational health were enlisted to present real-practice problems during the IPE activity. Additionally, they shared example case studies illustrating interprofessional collaborative practices. These professionals encouraged participants in the IPE activity to collaborate in thinking of solutions for problems drawn from their respective practices. They also engaged in reflective discussions with students on interprofessional approaches to occupational health issues. Furthermore, these professionals were involved in course development and delivery, provided coaching to participants throughout the course, and offered feedback on presentations. Their involvement contributed to the successful execution of in-class learning activities such as case studies [39].

Moreover, highly motivated and knowledgeable educators, who conveyed their enthusiasm to the participants, contributed to the overall success of the IPE activity [52]. The enthusiasm exhibited by the educators during the training sessions contributed to the motivation of participants to apply the acquired knowledge in their daily practice [52]. In another study, assistance of experienced faculty members facilitated comprehensible and effective knowledge transfer among participants from diverse disciplines [53]. During the IPE activity, participants presented their findings on occupational health and safety within specific working conditions. The involved faculty members supported participants in making the presented information accessible to all participating disciplines. Gillespie et al. [54] suggested that interaction with field specialists helped students connecting with the field and diverse disciplines within occupational health and safety. Additionally, this interaction helped students identify employment and career possibilities [54].

Lastly, another study showed the importance of involving experts such as faculty, alumni, and an external advisory board in the planning and organization of an IPE activity [54]. Faculty were responsible for identification of suitable sites to visit during interprofessional field trips. This process was facilitated by the participation of alumni of the involved educational programs and an external advisory board with the involved Educational and Research Center. The advisory board provided a comprehensive understanding of all participating educational programs. The involvement of these experts facilitated the selection of work sites that appealed to a diverse range of occupational health and safety disciplines [54].

**Interaction possibilities**. One study highlighted the importance of promoting interprofessional approaches to occupational health and safety issues at the workplace by enhancing (informal) interaction between participants of the IPE activity (i.e., students from different work-focused healthcare professions), and between participants and faculty from different disciplines [53]. Sufficient interaction time should be incorporated into the IPE activity [53]. Interaction can extend beyond the formal course activities. Two studies, in which the IPE activity consisted of work site visits, proper logistics (such as joint transportation, shared meals, and overnight stays at the same hotel) facilitated informal discussions among participants from various disciplines [53, 54]. For example, the bus used to transport participants to work sites served not only as transportation, but also provided the opportunity for reflection on observations and collaboration on group assignments [53, 54]. Interaction with field experts occurred during formal presentations, but also during site tours and informal meals [53, 54]. The ability and opportunity to interact with professionals from different disciplines supported the IPE activity’s objective of promoting interprofessional collaboration in addressing complex occupational health problems [53].

**Reflection possibilities**. Gillespie et al. [54] emphasized the importance of reflection opportunities for successful IPE activities. Following their participation in an interprofessional field trip, students engaged in structured discussions with faculty members. During these discussions, students connected their interprofessional field trip experiences to the materials they had learned in their educational program. Through discussion and reflection, students were able to rectify assumptions about other disciplines and their tasks and responsibilities. Reflective discussions took place shortly after the interprofessional field trip, for example during group dinner or on the bus ride home.

### Barriers related to the characteristics of the IPE activity

**Inadequate alignment with previous experience, knowledge, and education.** One study suggested that lack of alignment with previous experience, knowledge, and education was a barrier to successful achievement of the goals of the IPE activity [54]. Gillespie et al. [54] developed interprofessional field trips where students from various disciplines explored the intersection of multiple occupational health and safety fields within a real-world setting. During the evaluation, some of the students expressed that the IPE program should be more closely aligned with their primary, monoprofessional educational program [54].

**Perceived lack of added value.** One study highlighted the importance of the IPE activity having added value above existing knowledge [51]. The aim of the IPE activity in this study was to enhance collaborative behavior between general practitioners and occupational health physicians in supporting sick workers with low back pain. Despite shifts in attitudes toward each other’s professions, the outcomes revealed no alteration in the collaborative behavior of the physicians involved. This lack of change may have resulted from the general practitioner’s belief that their practices already aligned with the guidelines for treating low back pain, which impeded efforts to improve management of low back pain. Consequently, the IPE activity failed to add value for the participants and did not alter the professionals’ behavior [51].

### Facilitators related to the characteristics of the participant

**Personal qualifications.** Several specific personal qualifications of participants of the IPE activity were mentioned as facilitating factors for the success of IPE activities [50]. These qualifications include leadership and commitment. In one of the papers, it was mentioned that collaboration fostered by IPE in work-focused healthcare can especially be supported by social competences of the occupational physician that go beyond their employer’s interests [50]. A professional might have their own norms and values and in order to achieve a successful IPE activity, it is important to be mindful of those norms and experiences and to incorporate them in the IPE activity, such as different language use [50]. However, it should be noted that this study has low methodological quality and the result should therefore be interpreted with caution.

### Barriers related to the characteristics of the participant

**Insufficient group interaction skills and commitment.** One study stated that participants of an IPE activity need the required communication skills in order to interact with other participants during the IPE activity [50]. Lower levels of communication skills to express oneself in a group could impact group dynamics during the IPE activity. It was noted that cooperative behavior to be open to interact with others can be influenced by effective verbal communication skills. An ingredient to achieve this was noted as the commitment to each other and empathy. However, it should be noted that this study has low methodological quality and the result should therefore be interpreted with caution.

Moreover, language barriers hindered effective IPE. During an international IPE activity described in one of the studies [52], some of the participants were not confident enough to speak English or had difficulty following the IPE activity. As a result, parts of the presentation and questions asked by non-English speakers had to be translated. This hindered the workflow of the IPE activity and resulted in prolongation of lectures [52].

**Misunderstanding of the role of other participants**. One study suggested that misunderstanding of other participants’ roles and views could hinder interprofessional collaboration [51]. Therefore, the IPE activity should provide attention to understanding each other’s role and vision. In the IPE activity of this study, general practitioners and occupational physicians learned to work with a protocol that indicated desired moments and context for collaboration in treatment of workers with low back pain. During the IPE activity, participants learned to work together using this protocol. In this way, participants could learn about each other’s roles and views.

### Facilitators related to the characteristics of the organizational context

**Practical applicability.** Authors of one study suggested that the possibility to put learned knowledge into practice immediately could facilitate the goals of the IPE activity [49]. For example, a joint vocational training is expected to have more impact on interprofessional collaboration compared to an undergraduate course [49].

**Repeated IPE experiences**. Studies highlighted the importance of repeating the IPE experience to increase interprofessional opportunities for participants [39, 53]. One study recommended not to limit learning about IPE competencies to one single course, but to incorporate IPE in multiple activities program-wide [39]. For example by encouraging interprofessional collaboration during field trips. Another study created a second version of the initial IPE activity, which was similar in structure but different in content [53]. In this way, participants could also follow the IPE activity the subsequent year of their educational degree.

**Hands-on practical experiences and adequate logistics**. Two IPE activities that contained visits to several work sites showed that willingness of work sites to participate in the IPE activity and adequate logistics (e.g., organized itinerary, adequate transportation) are important [53, 54]. Identification of suitable work sites in which all work-focused health disciplines are encountered, the possibility to visit these work sites, an organized itinerary, and the desire of these work sites to participate in the training facilitated effective IPE in terms of understanding occupational health from an interprofessional perspective [53, 54]. Moreover, a hands-on experience at the work site including the possibility to engage with personnel that guided and informed the participants facilitated opportunities to learn about occupational hazards and control [53] and helped students to identify differences between expectations and reality [54].

### Barriers related to the characteristics of the organizational context

**Time-related challenges.** Several studies mentioned time-related challenges regarding the execution and effectiveness of the IPE activity [39, 49, 50]. Being a one-credit, undergraduate course that also contained other topics resulted in limited time for the actual IPE activities [39]. This hindered acquisition of IPE competencies. Additional, outside-class IPE activities, such as students interacting with each other to better understand each other’s discipline, discussing occupational safety issues with faculty, and working on group assignments outside class helped to overcome these time-related issues. However, it is advised to allocate additional class time for these activities [39].

In addition, one study showed that participating in a one-time IPE activity did not affect outcomes in the long-term. With their IPE activity, Nauta et al. aimed to increase trust between participants from various disciplines [49]. Directly after the IPE activity, participants reported increased trust in participants from other disciplines. However, reported trust before the IPE activity and three months after the IPE activity did not differ. More frequent and structural activities were suggested to change attitudes and behavior sustainably [49].

**Lack of organizational support**. Lack of organizational support was reported as a barrier to effective IPE [50]. However, it should be noted that this barrier is based only on the study with low methodological quality and should therefore be interpreted with caution. For effective IPE trainings to be developed, organizational support in terms of resources and allocated time for professionals to participate was mentioned as essential. However, most companies where occupational physicians are employed do not allocate dedicated time during working hours for IPE activities and continuing education [50].

The same study reported lack of collaboration between the educational academy, universities, and other specialized institutes as a barrier to effective IPE [50]. It was noted that effective IPE activities should be developed in collaboration with universities, because they offer the necessary academic background and theoretical knowledge to incorporate into the IPE activities. These activities could then be implemented in companies where occupational physicians work and collaborate with other professionals.

**Restrictions due to organizational regulations.** Organizational policies and restrictions were found to be barriers to effective IPE. One study mentioned that course format affected the IPE activity [39]. Due to COVID-19 regulations of the university where the IPE activity was offered, the format changed from at location to online. The study authors suggested that following the program online might have hindered the IPE activity’s goal of understanding each other’s roles.

For another IPE activity, professionals were dependent on a scholarship to be able to participate in the IPE activity. However, only professionals who were affiliated to partner universities and followed part of their education in the country of the IPE activity were eligible to receive a scholarship [52]. This was not only deemed unfair by prospective participants, but this selection bias could also have hindered the IPE activity’s goal of participants acquiring knowledge on occupational health in a global context.

### Facilitators related to the characteristics of the socio-political context

**Supported by law.** The course being based on a lawful act was found to be a facilitator for IPE [50]. However, it should be noted that this facilitator was based on the study with low methodological quality and should therefore be interpreted with caution. The study mentioned that that lawful acts supporting interprofessional collaboration can support the development and provision of IPE activities in terms of funding and uptake in practice. In this specific study, the IPE activity was developed in line with a lawful act that encourages interprofessional collaborative practice, which contributes to a broader urgency to adopt skills as well as support for following the IPE activity.

### Barriers related to characteristics of the socio-political context

**Lack of support from external organizations.** One study reported a lack of support from external organizations (e.g., companies and work sites to be visited as part of the IPE activity) as a barrier to effective IPE [53]. For example, participants from one IPE activity were not allowed to visit a suitable work site due to physical safety concerns that restricted access to that work site [53]. As a result, participants could not experience the multidisciplinary approach to occupational health that was practiced at this work site.

## Discussion

The aim of this integrative review was to identify, summarize, and synthesize barriers and facilitators for IPE for work-focused healthcare professionals. Barriers and facilitators were identified across four levels based on the MIDI framework [45]: characteristics of the IPE activity, characteristics of the participants, characteristics of the organizational context, and characteristics of the socio-political context. Findings related to the characteristics of the IPE activity reveal several important factors: the perceived added value of the IPE activity [51], the involvement of skilled and dedicated educators who effectively connect course material to work-focused healthcare practice in both the development and the execution of the IPE activity [39, 52–54], and the availability of sufficient opportunities for formal and informal interaction, as well as the reflection on the IPE experience and each other’s roles and responsibilities both during and after the IPE activity [53, 54]. Regarding the characteristics of the participants in the IPE activity, lack of interaction skills and misunderstandings about roles of other participating professionals were found to be barriers [50–52]. Presence of personal qualifications such as social competences and being mindful of norms and values was found to be a facilitator [50]. Most barriers and facilitators at the level of organizational context characteristics revolved around several forms of organizational support [39, 49, 50, 52, 53]. For example, in terms of time and resources allocated to the IPE activity, facilitators included the opportunity for participants to engage in hands-on, practical experiences [52–54]. A barrier regarding characteristics of the socio-political context was lack of support from external organizations. For this level, the IPE activity being supported by a lawful act was identified as a facilitator [50].

The findings of this review show the importance of linking the IPE activity to the daily practice of work-focused healthcare professionals. Involving educators who are skilled in both IPE and work-focused healthcare in the development and facilitation of these activities can convey enthusiasm for interprofessional collaboration in participants’ own work practice [39, 52–54]. These educators can effectively bridge the gap between IPE concepts and their practical application in work settings. The value of the IPE activity for one’s daily practice should be clear, otherwise it may hinder changes in interprofessional collaborative behavior [51]. To help connect education to practice, the IPE activity can be designed as vocational training or include site visits [49, 52–54]. This could also be underpinned by the socio-cultural learning theory, that argues that learning takes place in a social context [55]. As our findings suggest, the social context of the IPE activity should be connected to the daily practice of work-focused healthcare professionals. This aligns with the educational perspective which emphasizes the importance of creating connectivity between education and practice. As work-focused healthcare practice is continuously evolving [4], learning and working become increasingly interdependent [56]. Therefore, IPE should be designed to integrate with work practices [56].

The findings of this review also show the importance of incorporating the training of interprofessional collaborative skills in the IPE activity. For example, when participants misunderstood the role of other participants, interprofessional collaboration during the IPE activity was hindered [51]. Meanwhile, personal competences and values such as commitment to interprofessional collaboration and social skills seemed to facilitate IPE [50]. The IPE activity should contain sufficient opportunities to develop these skills, for example, by integrating interaction and reflection possibilities into its design to foster social and reflective competencies [53, 54]. The necessity to train for interprofessional skills identified in this review aligns with a previously established competency set for IPE: the Interprofessional Education Collaborative (IPEC) Core Competencies for Interprofessional Collaborative Practice [57]. This set of competencies can be used to guide IPE for both students and professionals, aiming to prepare health professionals to collaborate intentionally and effectively in building a safe, person-centered, and population-oriented health care system [57, 58]. The set consists of four core competencies: 1) value/ethics for interprofessional practice, 2) roles and responsibilities, 3) interprofessional communication, and 4) teams and teamwork. This review shows that the first three competencies are acknowledged in research on IPE in the field of work-focused healthcare. However, the competency ‘teams and teamwork’ was not found in the studies included in this review. This could be because research on IPE in the field of work-focused healthcare is still limited. However, it is also possible that work-focused healthcare professionals do not often work in fixed teams, which may affect their experience of collaboration in a team setting. As a result, training the competency ‘teams and teamwork’ might initially seem less relevant in an work-focused IPE activity. In relation to this, the variety of professions that participated in the studies included in this review suggests that interprofessional collaboration in the field of work-focused healthcare encompasses a range of stakeholders that does not fit a traditional, fixed team. Thus, in the context of RTW processes, the team may need to extend beyond healthcare professionals to include other important stakeholders, such as employers, human resources (HR) professionals, insurance representatives, and social workers. Providing RTW support can be seen as a societal issue requiring input from diverse disciplines, including those beyond traditional healthcare professions [59]. As such, interprofessional collaboration may vary depending on the case and context [60]. For example, in the Dutch context, the occupational physician, social insurance physician, and labor expert are seen as the most prominent stakeholders in the RTW process. However, in other countries, RTW is organized differently [8, 29]. Moreover, each unique case could benefit from collaboration with other (allied) health professionals, such as psychologists, occupational health nurses, or rehabilitation counselors. These diverse teams highlight the need for IPE to incorporate flexibility and adapt to the specific stakeholders relevant to each case and context.

The findings of this review also indicate that, to effectively acquire these interprofessional competencies, sufficient time for IPE activities should be integrated into the curriculum for both work-focused healthcare students and the continuing education of professionals [39]. Moreover, a lack of sustained changes in attitudes toward interprofessional collaboration over time suggests that structural IPE experiences are necessary [49]. Therefore, it is argued that IPE in work-focused healthcare should be implemented as part of a learning continuum, spanning from foundational education to continuing professional development [48]. However, foundational, undergraduate curricula often place little emphasis on work-focused healthcare [61]. For example, most medical undergraduate curricula include minimal content on work-focused healthcare, with more specialized training, such as for occupational physicians and social insurance physicians, occurring at the postgraduate level. At the same time, there is an increasing need for other (healthcare) professionals to be informed about work-focused healthcare in their daily practice [62]. For instance, previous research has shown that patients often feel the need to discuss issues related to work and health with the involved professionals throughout their work-focused healthcare trajectory, for instance medical specialists, psychologists or rehabilitation professionals [63–65]. This raises the question of whether more focus should be placed on work-focused healthcare in undergraduate (medical) education in general and on how IPE can play a role in this. It can also be argued that in foundational education, IPE can be more general, focusing on the acquisition of interprofessional competencies. These competencies can be further applied and expanded in graduate education and continuing professional development, particularly in the context of work-focused healthcare. Moreover, the field of work-focused healthcare is continuously evolving [4], making lifelong learning essential for adapting to these changes [56]. As this review also indicates, organizational and legal support are key to enabling integration of IPE into a learning continuum. For example, accreditation bodies often mandate the inclusion of IPE in undergraduate programs to a varying extent, though the focus is generally broad and not specific to work-focused healthcare [57, 66]. Support mechanisms such as allocating time for IPE, providing funding for IPE, or promoting the uptake of IPE can facilitate its acknowledgement and integration into both undergraduate and graduate curricula, as well as continuing professional development [50].

The current review also highlights the role of reflection in the development of interprofessional competencies [54]. This aligns with previous research on reflection in education. IPE and interprofessional collaboration occur in diverse situations, requiring reflective practitioners who continuously improve their skills through self-reflection on their interprofessional performance [67]. Through reflection, learners can analyze their interprofessional experiences, recognize their own assumptions and beliefs toward other professionals and their own behavior, and understand how these assumptions can impact their decisions regarding interprofessional collaboration [68]. Reflection can be incorporated in IPE in two ways: through reflection-in-action and through reflection-on-action [67, 69]. During reflection-in-action, participants reflect on what they are doing, while they are doing it. They compare newly acquired knowledge with what is already known. This form of reflection helps to create abstract concepts and to make sense of new data [70]. Reflection-on-action, on the other hand, occurs after the IPE activity. Participants then reflect on their actions in the IPE activity. This retrospective analysis helps participants understand how learning outcomes came about [69, 70]. Thus, interprofessional collaboration requires a reflective practitioner to handle situations in which interprofessional collaboration is required.

## Strengths and limitations

It is recommended that the findings of this integrative review be interpreted with caution due to several limitations. The first limitation stems from differences in description outcome measures and context factors of the included studies. The studies assessed various learning and health outcomes that were mostly self-reported by participants. This complicates the comparison of barriers and facilitators across included studies, as they affect different aspects of the IPE activity. Even when similar outcome measures were used, different instruments were used for data collection which may hinder comparison of the results across studies. Moreover, replicability and sustainability of an IPE activity can be supported by data on context factors and learning and teaching processes [71]. However, this kind of data was often not described in the included studies. When interpreting results, it is important to consider which aspect of the IPE activity each barrier or facilitator affects and how context factors play into this. To address this limitation, future studies should use a more comprehensive approach for evaluation of their IPE activity. For example, the realist evaluation approach [72].

The second limitation relates to methodological aspects. First, this review included only seven eligible studies on IPE in work-focused healthcare. The discussed barriers and facilitators are thus based on a limited number of studies which varied in both study design and outcome measures. This limited availability of studies on IPE for work-focused healthcare professionals illustrates the need for the development of new IPE activities in this field. Moreover, only studies published in English, Dutch or German were included in this review. This could have introduced language bias. However, previous research suggests no systematic bias through the use of language constraints in systematic reviews [73].

Another methodological issue that should be taken into account is the methodological quality of included studies. One study had low methodological quality [50]. Nevertheless, it was decided not to exclude this study based on its quality assessment score, as it still provided valuable insights into its IPE activity and therefore contained important information regarding the barriers and facilitators for IPE. However, it is suggested that the findings coming from this study which are not supported by other studies (i.e., facilitator ‘personal qualifications’ and facilitator ‘based on a lawful act’) are interpreted with caution. Future research is necessary to further explore the potential enabling effects these facilitators may have on IPE.

A strength of this integrative review is the use of a broad search strategy to identify eligible studies. This approach resulted in a large number of studies for the initial screening phase (i.e., title screening). This broad search strategy was essential for two reasons: 1) IPE activities are diverse and are therefore described using various terms, and 2) terms indicating ‘interprofessional’ are often used incorrectly and/or interchangeably [18, 19]. For example, some studies refer to multidisciplinary education, while their educational activity is in fact interprofessional. This distinction is important, as multidisciplinary collaboration implies information sharing between professionals while working alongside each other with separate goals [18, 20]. The focus is on accomplishing individual tasks rather than on the collective process [18]. In contrast, interprofessional collaboration involves the joint setting and evaluation of goals, active interaction among professionals from different disciplines, and the exchange of knowledge and competencies [18, 20]. Since these terms are often used interchangeably in studies on interprofessional education, it was important for the search strategy of this review to be sensitive to all these terms to identify all relevant articles. During the screening stage, the authors verified whether the IPE activity was interprofessional rather than multiprofessional or multidisciplinary in nature.

### Implications for future research and practice

Following the above mentioned issues, several recommendations for future IPE development and research are suggested. First, more IPE development and subsequent research in the field of work-focused healthcare is needed. This review identified only seven studies that described the development or evaluation of an IPE activity targeted at (at least one group of) work-focused healthcare professionals. Additional research is particularly necessary to investigate the effectiveness of IPE on interprofessional practice. Measures of effectiveness should therefore go beyond course evaluations, and instead focus on more distant outcome measures, such as interprofessional collaboration and its impact on patient outcomes in practice [74]. Health and system outcomes (e.g., individual health outcomes, organizational change outcomes) are especially underrepresented in IPE research [19]. It is recommended to use existing, formal measures such as validated questionnaires to measure outcomes of IPE activities [75]. For example, at the participant level, measures such as the Readiness for Interprofessional Learning Scale [76, 77] or the Interdisciplinary Education Perception Scale [78, 79] could be used. In addition, further studies in this field are encouraged to avoid using terms such as ‘interprofessional,’ ‘multiprofessional,’ ‘interdisciplinary,’ and ‘multidisciplinary’ interchangeably or incorrectly. It is recommended to carefully define what the educational activity should accomplish and use the appropriate term to describe this.

Second, none of the included studies described a connection between (learning) theory and their IPE activity. Without the use of theory, assumptions on why and how phenomena occur cannot be tested [80]. Theory contributes to the design, delivery, and evaluation of an IPE activity [80, 81]. Moreover, theory helps to explain the underpinnings of an IPE activity [82]. Therefore, it is suggested that future endeavors of IPE in work-focused healthcare integrate theory and established education design principles in their curriculum design, development, and evaluation. Moreover, future research is encouraged to establish a more overarching theory on why and how interprofessional collaborative practice in the field of work-focused healthcare can be shaped by using IPE.

## Conclusion

This review identified barriers and facilitators for IPE aimed at work-focused healthcare professionals. Insight into these barriers and facilitators can guide and stimulate future development of IPE activities targeted at this field of practice. Future IPE endeavors are encouraged to incorporate educational design principles and learning theories into their curriculum development. IPE for work-focused healthcare professionals should be integrated in their learning continuum, should be connected to professionals’ daily practice, and should include training of interprofessional competencies and reflective skills. Future research is needed to assess the impact of IPE activities on interprofessional practice, as well as on health and patient outcomes.

## Supplementary Information

Below is the link to the electronic supplementary material.

Supplementary file1 (DOCX 40 KB)

Supplementary file2 (DOCX 33 KB)

Supplementary file3 (XLSX 18 KB)

Supplementary file4 (XLSX 27 KB)

## Data Availability

The datasets generated and/or analyzed during the current study are available from the corresponding author on reasonable request.
