# Supplementary material for: Barriers and Facilitators for Interprofessional Education in Work-Focused Healthcare: An Integrative Review
Source: J Occup Rehabil. 2025 Feb 23;36(1):97–116. doi: 10.1007/s10926-025-10278-3 (PMC12906514; doi:10.1007/s10926-025-10278-3)
Supplement: Supplementary file 2 — Supplementary file2 (DOCX 33 KB) [file 10926_2025_10278_MOESM2_ESM.docx]

**Online Resource 2: Search strategy per database**

**Barriers and facilitators for interprofessional education in work-focused healthcare: an integrative review**

Journal of Occupational Rehabilitation

Elmi Zwaan, Nina Zipfel, Wietske Kuijer-Siebelink, Shirley Oomens, Sylvia J. van der Burg – Vermeulen

**Corresponding author**

Elmi Zwaan

Amsterdam UMC, Department of Public and Occupational Health, Amsterdam Public Health research institute, Amsterdam, The Netherlands.

Email: [e.zwaan1@amsterdamumc.nl](mailto:e.zwaan1@amsterdamumc.nl)

CINAHL

S1 MH "Education, Interdisciplinary" OR TI(((Cross OR collaborative OR cooperat* OR "co operat*" OR interprofessional* OR interdisciplinar* OR multidisciplinar* OR crossdisciplinar* OR crossprofession* OR intraprofessional* OR multiprofessional* OR transdisciplin*) N1 (training* OR education*)) OR (joint N1 vocation* N1 train* N1 program*) OR ((inter OR intra OR multi OR trans OR cross ) N1 (professional* OR disciplinar*) N1 (training* OR education*))) OR AB(((Cross OR collaborative OR cooperat* OR "co operat*" OR interprofessional* OR interdisciplinar* OR multidisciplinar* OR crossdisciplinar* OR crossprofession* OR intraprofessional* OR multiprofessional* OR transdisciplin*) N1 (training* OR education*)) OR (joint N1 vocation* N1 train* N1 program*) OR ((inter OR intra OR multi OR trans OR cross ) N1 (professional* OR disciplinar*) N1 (training* OR education*))) OR SU(((Cross OR collaborative OR cooperat* OR "co operat*" OR interprofessional* OR interdisciplinar* OR multidisciplinar* OR crossdisciplinar* OR crossprofession* OR intraprofessional* OR multiprofessional* OR transdisciplin*) N1 (training* OR education*)) OR (joint N1 vocation* N1 train* N1 program*) OR ((inter OR intra OR multi OR trans OR cross ) N1 (professional* OR disciplinar*) N1 (training* OR education*)))

S2 MH "Multidisciplinary Care Team" OR TI(interprofessional* OR interdisciplinar* OR multidisciplinar* OR crossdisciplinar* OR crossprofession* OR intraprofessional* OR multiprofessional* OR ((inter OR intra OR multi OR trans OR cross) W1 (professional* OR disciplinar*)) OR transdisciplin* OR (collaborative W1 (care OR healthcare) W1 team*)) OR SU(interprofessional* OR interdisciplinar* OR multidisciplinar* OR crossdisciplinar* OR crossprofession* OR intraprofessional* OR multiprofessional* OR ((inter OR intra OR multi OR trans OR cross) W1 (professional* OR disciplinar*)) OR transdisciplin* OR (collaborative W1 (care OR healthcare) W1 team*))

S3 MH "Education+" OR MW "ED" OR TI(educat* OR cours* OR training OR teach*)

S4 S2 AND S3

S5 S1 OR S4

S6 MH "Occupational Health" OR MH "Impairment, Health Professional" OR MH "Stress, Occupational+" OR MH "Occupational Medicine" OR MH "Occupational Diseases" OR TI(((employee* OR work OR insurance* OR occupational OR job) W0 (medicin* OR health* OR stress)) OR (Employ* N1 Assistance N1 Program*) OR (employee* N1 Based N1 Service*) OR OSH OR (Occupational N1 safety) OR ((occupational OR worker* OR professional) W0 (health* OR fitness))) OR AB(((employee* OR work OR insurance* OR occupational OR job) W0 (medicin* OR health* OR stress)) OR (Employ* N1 Assistance N1 Program*) OR (employee* N1 Based N1 Service*) OR OSH OR (Occupational N1 safety) OR ((occupational OR worker* OR professional) W0 (health* OR fitness))) OR SU(((employee* OR work OR insurance* OR occupational OR job) W0 (medicin* OR health* OR stress)) OR (Employ* N1 Assistance N1 Program*) OR (employee* N1 Based N1 Service*) OR OSH OR (Occupational N1 safety) OR ((occupational OR worker* OR professional) W0 (health* OR fitness)))

S7 MH "Occupational Health Services+" OR TI(((Compan* OR occupation* OR insurance OR OH OR industrial) W1 (Physician* OR doctor* OR practitioner* OR nurs*)) OR ((labor* OR labour* OR employment OR job) N1 (expert* OR official OR officials OR consultant*))) OR AB(((Compan* OR occupation* OR insurance OR OH OR industrial) W1 (Physician* OR doctor* OR practitioner* OR nurs*)) OR ((labor* OR labour* OR employment OR job) N1 (expert* OR official OR officials OR consultant*))) OR SU(((Compan* OR occupation* OR insurance OR OH OR industrial) W1 (Physician* OR doctor* OR practitioner*)) OR ((labor* OR labour* OR employment OR job) N1 (expert* OR official OR officials OR consultant*)))

S8 MH "Job Re-Entry" OR TI(((return* OR back) N1 to N1 (work OR job* OR labor* OR labour* OR occupation* OR vocation* OR employment)) OR ((work OR worker* OR job* OR labor* OR labour* OR vocation* OR employment OR personnel) N1 (resum* OR reintegrat* OR reentr* OR "re-entry" OR retention OR participat* OR rehab*)) OR (occupation* N0 (resum* OR reintegrat* OR reentr* OR "re-entry" OR retention OR participat* OR rehab*)) OR (stay* N1 at N1 (work OR job* OR labor* OR labour* OR occupation* OR vocation* OR employment)) OR employabilit* OR employable) OR AB(((return* OR back) N1 to N1 (work OR job* OR labor* OR labour* OR occupation* OR vocation* OR employment)) OR ((work OR worker* OR job* OR labor* OR labour* OR vocation* OR employment OR personnel) N1 (resum* OR reintegrat* OR reentr* OR "re-entry" OR retention OR participat* OR rehab*)) OR (occupation* N0 (resum* OR reintegrat* OR reentr* OR "re-entry" OR retention OR participat* OR rehab*)) OR (stay* N1 at N1 (work OR job* OR labor* OR labour* OR occupation* OR vocation* OR employment)) OR employabilit* OR employable) OR SU(((return* OR back) N1 to N1 (work OR job* OR labor* OR labour* OR occupation* OR vocation* OR employment)) OR ((work OR worker* OR job* OR labor* OR labour* OR vocation* OR employment OR personnel) N1 (resum* OR reintegrat* OR reentr* OR "re-entry" OR retention OR participat* OR rehab*)) OR (occupation* N0 (resum* OR reintegrat* OR reentr* OR "re-entry" OR retention OR participat* OR rehab*)) OR (stay* N1 at N1 (work OR job* OR labor* OR labour* OR occupation* OR vocation* OR employment)) OR employabilit* OR employable)

S9 MH "Sick Leave" OR MH "Absenteeism" OR MH "Presenteeism" OR MH "Productivity" OR MH "Job Performance" OR MH "Personnel Retention" OR TI(((sick* OR disability* OR medical) N1 (leave* OR absen*)) OR ((sick OR illness) N1 day*) OR Absenteeism OR presenteeism OR presenteism OR (sickness N1 presence) OR (working N1 while N1 (sick OR ill)) OR ((work OR worker* OR job* OR labor* OR labour* OR occupation* OR vocation* OR employ* OR personnel) N1 (absen* OR disab* OR disease* OR illness OR impair* OR productivit* OR perform* OR particip* OR abilit* OR loss)) OR Presenteeism OR underemploy* OR (under N1 employ*)) OR AB(((sick* OR disability* OR medical) N1 (leave* OR absen*)) OR ((sick OR illness) N1 day*) OR Absenteeism OR presenteeism OR presenteism OR (sickness N1 presence) OR (working N1 while N1 (sick OR ill)) OR ((work OR worker* OR job* OR labor* OR labour* OR occupation* OR vocation* OR employ* OR personnel) N1 (absen* OR disab* OR disease* OR illness OR impair* OR productivit* OR perform* OR particip* OR abilit* OR loss)) OR Presenteeism OR underemploy* OR (under N1 employ*)) OR SU(((sick* OR disability* OR medical) N1 (leave* OR absen*)) OR ((sick OR illness) N1 day*) OR Absenteeism OR presenteeism OR presenteism OR (sickness N1 presence) OR (working N1 while N1 (sick OR ill)) OR ((work OR worker* OR job* OR labor* OR labour* OR occupation* OR vocation* OR employ* OR personnel) N1 (absen* OR disab* OR disease* OR illness OR impair* OR productivit* OR perform* OR particip* OR abilit* OR loss)) OR Presenteeism OR underemploy* OR (under N1 employ*))

S10 (MH "Disability Management" OR TI((functional W1 (abilit* OR capacit*)) OR (disabilit* N0 manag*)) OR AB((functional W1 (abilit* OR capacit*)) OR (disabilit* N0 manag*)) OR SU((functional W0 (abilit* OR capacit*)) OR (disabilit* N0 manag*))) AND (MH "Work+" OR TI(work OR worker* OR job* OR labor* OR labour* OR occupation* OR vocation* OR employ* OR personnel))

S11 S6 OR S7 OR S8 OR S9 OR S10

S12 S5 AND S11

COCHRANE

#1 (((Cross OR collaborative OR cooperat* OR "co operat*" OR interprofessional* OR interdisciplinar* OR multidisciplinar* OR crossdisciplinar* OR crossprofession* OR intraprofessional* OR multiprofessional* OR transdisciplin*) NEAR/1 (training* OR education*)) OR (joint NEAR/1 vocation* NEAR/1 train* NEAR/1 program*)):ti,ab,kw

#2 ((inter OR intra OR multi OR trans OR cross ) NEAR/1 (professional* OR disciplinar*) NEAR/1 (training* OR education*)):ti,ab,kw

#3 (interprofessional* OR interdisciplinar* OR multidisciplinar* OR crossdisciplinar* OR crossprofession* OR intraprofessional* OR multiprofessional* OR ((inter OR intra OR multi OR trans OR cross) NEAR/1 (professional* OR disciplinar*)) OR transdisciplin* OR (collaborative NEAR/1 (care OR healthcare) NEAR/1 team*)):ti,ab,kw

#4 (educat* OR cours* OR training OR teach*):ti

#5 #3 AND #4

#6 #1 OR #2 OR #5

#7 (((employee* OR work OR insurance* OR occupational OR job) NEAR/1 (medicin* OR health* OR stress)) OR (Employ* NEAR/1 Assistance NEAR/1 Program*) OR (employee* NEAR/1 Based NEAR/1 Service*) OR OSH OR (Occupational NEAR/1 safety) OR ((occupational OR worker* OR professional) NEXT/0 (health* OR fitness))):ti,ab,kw

#8 (((Compan* OR occupation* OR insurance OR OH OR industrial) NEXT/1 (Physician* OR doctor* OR practitioner* OR nurs*)) OR ((labor* OR labour* OR employment OR job) NEAR/1 (expert* OR official OR officials OR consultant*))):ti,ab,kw

#9 (((return* OR back) NEAR/1 to NEAR/1 (work OR job* OR labor* OR labour* OR occupation* OR vocation* OR employment)) OR ((work OR worker* OR job* OR labor* OR labour* OR vocation* OR employment OR personnel) NEAR/1 (resum* OR reintegrat* OR reentr* OR "re-entry" OR retention OR participat* OR rehab*)) OR (occupation* NEXT/0 (resum* OR reintegrat* OR reentr* OR "re-entry" OR retention OR participat* OR rehab*)) OR (stay* NEAR/1 at NEAR/1 (work OR job* OR labor* OR labour* OR occupation* OR vocation* OR employment)) OR employabilit* OR employable):ti,ab,kw

#10 (((sick* OR disability* OR medical) NEAR/1 (leave* OR absen*)) OR ((sick OR illness) NEAR/1 day*) OR Absenteeism OR presenteeism OR presenteism OR (sickness NEAR/1 presence) OR (working NEAR/1 while NEAR/1 (sick OR ill)) OR ((work OR worker* OR job* OR labor* OR labour* OR occupation* OR vocation* OR employ* OR personnel) NEAR/1 (absen* OR disab* OR disease* OR illness OR impair* OR productivit* OR perform* OR particip* OR abilit* OR loss)) OR Presenteeism OR underemploy* OR (under NEAR/1 employ*)):ti,ab,kw

#11 ((functional NEXT/1 (abilit* OR capacit*)) OR (disabilit* NEAR/0 manag*)):ti,ab,kw AND (work OR worker* OR job* OR labor* OR labour* OR occupation* OR vocation* OR employ* OR personnel):ti

#12 #7 OR #8 OR #9 OR #10 OR #11

#13 #6 AND #12

EMBASE

#1 'interdisciplinary education'/de OR (((Cross OR collaborative OR cooperat* OR "co operat*" OR interprofessional* OR interdisciplinar* OR multidisciplinar* OR crossdisciplinar* OR crossprofession* OR intraprofessional* OR multiprofessional* OR transdisciplin*) NEAR/1 (training* OR education*)) OR (joint NEAR/1 vocation* NEAR/1 train* NEAR/1 program*)):ti,ab,kw

#2 ((inter OR intra OR multi OR trans OR cross ) NEAR/1 (professional* OR disciplinar*) NEAR/1 (training* OR education*)):ti,ab,kw

#3 'multidisciplinary team'/de OR 'collaborative care team'/de OR (interprofessional* OR interdisciplinar* OR multidisciplinar* OR crossdisciplinar* OR crossprofession* OR intraprofessional* OR multiprofessional* OR ((inter OR intra OR multi OR trans OR cross) NEAR/1 (professional* OR disciplinar*)) OR transdisciplin* OR (collaborative NEAR/1 (care OR healthcare) NEAR/1 team*)):ti,ab,kw

#4 'education'/exp OR (educat* OR cours* OR training OR teach*):ti

#5 #3 AND #4

#6 #1 OR #2 OR #5

#7 'occupational health'/de OR 'occupational health nursing'/de OR 'job stress'/de OR 'occupational medicine'/de OR (((employee* OR work OR insurance* OR occupational OR job) NEAR/1 (medicin* OR health* OR stress)) OR (Employ* NEAR/1 Assistance NEAR/1 Program*) OR (employee* NEAR/1 Based NEAR/1 Service*) OR OSH OR (Occupational NEAR/1 safety) OR ((occupational OR worker* OR professional) NEXT/0 (health* OR fitness))):ti,ab,kw

#8 'occupational health service'/de OR (((Compan* OR occupation* OR insurance OR OH OR industrial) NEXT/1 (Physician* OR doctor* OR practitioner* OR nurs*)) OR ((labor* OR labour* OR employment OR job) NEAR/1 (expert* OR official OR officials OR consultant*))):ti,ab,kw

#9 'vocational rehabilitation'/de OR 'employability'/de OR 'work resumption'/de OR 'return to work'/exp OR (((return* OR back) NEAR/1 to NEAR/1 (work OR job* OR labor* OR labour* OR occupation* OR vocation* OR employment)) OR ((work OR worker* OR job* OR labor* OR labour* OR vocation* OR employment OR personnel) NEAR/1 (resum* OR reintegrat* OR reentr* OR "re-entry" OR retention OR participat* OR rehab*)) OR (occupation* NEXT/0 (resum* OR reintegrat* OR reentr* OR "re-entry" OR retention OR participat* OR rehab*)) OR (stay* NEAR/1 at NEAR/1 (work OR job* OR labor* OR labour* OR occupation* OR vocation* OR employment)) OR employabilit* OR employable):ti,ab,kw

#10 'medical leave'/exp OR 'absenteeism'/exp OR 'presenteeism'/exp OR 'productivity'/exp OR 'job performance'/exp OR (((sick* OR disability* OR medical) NEAR/1 (leave* OR absen*)) OR ((sick OR illness) NEAR/1 day*) OR Absenteeism OR presenteeism OR presenteism OR (sickness NEAR/1 presence) OR (working NEAR/1 while NEAR/1 (sick OR ill)) OR ((work OR worker* OR job* OR labor* OR labour* OR occupation* OR vocation* OR employ* OR personnel) NEAR/1 (absen* OR disab* OR disease* OR illness OR impair* OR productivit* OR perform* OR particip* OR abilit* OR loss)) OR Presenteeism OR underemploy* OR (under NEAR/1 employ*)):ti,ab,kw

#11 ((functional NEXT/1 (abilit* OR capacit*)) OR (disabilit* NEAR/0 manag*)):ti,ab,kw AND ('work'/exp OR (work OR worker* OR job* OR labor* OR labour* OR occupation* OR vocation* OR employ* OR personnel):ti)

#12 #7 OR #8 OR #9 OR #10 OR #11

#13 #6 AND #12

ERIC

S1 TI(((Cross OR collaborative OR cooperat* OR "co operat*" OR interprofessional* OR interdisciplinar* OR multidisciplinar* OR crossdisciplinar* OR crossprofession* OR intraprofessional* OR multiprofessional* OR transdisciplin*) N1 (training* OR education*)) OR (joint N1 vocation* N1 train* N1 program*) OR ((inter OR intra OR multi OR trans OR cross ) N1 (professional* OR disciplinar*) NEAR/1 (training* OR education*))) OR AB(((Cross OR collaborative OR cooperat* OR "co operat*" OR interprofessional* OR interdisciplinar* OR multidisciplinar* OR crossdisciplinar* OR crossprofession* OR intraprofessional* OR multiprofessional* OR transdisciplin*) N1 (training* OR education*)) OR (joint N1 vocation* N1 train* N1 program*) OR ((inter OR intra OR multi OR trans OR cross ) NEAR/1 (professional* OR disciplinar*) NEAR/1 (training* OR education*))) OR SU(((Cross OR collaborative OR cooperat* OR "co operat*" OR interprofessional* OR interdisciplinar* OR multidisciplinar* OR crossdisciplinar* OR crossprofession* OR intraprofessional* OR multiprofessional* OR transdisciplin*) N1 (training* OR education*)) OR (joint N1 vocation* N1 train* N1 program*) OR ((inter OR intra OR multi OR trans OR cross ) NEAR/1 (professional* OR disciplinar*) NEAR/1 (training* OR education*)))

S2 DE "Interdisciplinary Approach" OR TI(interprofessional* OR interdisciplinar* OR multidisciplinar* OR crossdisciplinar* OR crossprofession* OR intraprofessional* OR multiprofessional* OR ((inter OR intra OR multi OR trans OR cross) NEAR/1 (professional* OR disciplinar*)) OR transdisciplin* OR (collaborative NEAR/1 (care OR healthcare) NEAR/1 team*)) OR AB(interprofessional* OR interdisciplinar* OR multidisciplinar* OR crossdisciplinar* OR crossprofession* OR intraprofessional* OR multiprofessional* OR ((inter OR intra OR multi OR trans OR cross) NEAR/1 (professional* OR disciplinar*)) OR transdisciplin* OR (collaborative NEAR/1 (care OR healthcare) NEAR/1 team*)) OR SU(interprofessional* OR interdisciplinar* OR multidisciplinar* OR crossdisciplinar* OR crossprofession* OR intraprofessional* OR multiprofessional* OR ((inter OR intra OR multi OR trans OR cross) NEAR/1 (professional* OR disciplinar*)) OR transdisciplin* OR (collaborative NEAR/1 (care OR healthcare) NEAR/1 team*))

S3 MH "Education+" OR DE "Education" OR DE "Academic Education" OR DE "Adult Education" OR DE "Adult Vocational Education" OR DE "Continuing Education" OR DE "Labor Education" OR DE "General Education" OR DE "Health Education" OR DE "Industrial Education" OR DE "Inservice Education" OR DE "Inservice Teacher Education" OR DE "Intergroup Education" OR DE "Nonformal Education" OR DE "Professional Education" OR DE "Medical Education" OR DE "Professional Continuing Education" OR DE "Vocational Education" OR DE "Adult Vocational Education" OR DE "Cooperative Education" OR DE "Prevocational Education" OR DE "Education Courses" OR DE "Learning" OR DE "Active Learning" OR DE "Adult Learning" OR DE "Associative Learning" OR DE "Aural Learning" OR DE "Authentic Learning" OR DE "Cooperative Learning" OR DE "Discovery Learning" OR DE "Electronic Learning" OR DE "Experiential Learning" OR DE "Game Based Learning" OR DE "Incidental Learning" OR DE "Intentional Learning" OR DE "Interference (Learning)" OR DE "Lifelong Learning" OR DE "Mastery Learning" OR DE "Multisensory Learning" OR DE "Nonverbal Learning" OR DE "Observational Learning" OR DE "Organizational Learning" OR DE "Prior Learning" OR DE "Problem Based Learning" OR DE "Rote Learning" OR DE "Sequential Learning" OR DE "Serial Learning" OR DE "Situated Learning" OR DE "Student Centered Learning" OR DE "Transfer of Training" OR DE "Transformative Learning" OR DE "Verbal Learning" OR DE "Visual Learning" OR DE "Workplace Learning" OR DE "Training" OR DE "Contract Training" OR DE "Job Training" OR DE "Professional Training" OR DE "Retraining" OR DE "Team Training" OR TI(educat* OR cours* OR training OR teach*)

S4 S2 AND S3

S5 S1 OR S4

S6 DE "Occupational Safety and Health" OR DE "Occupational Diseases" OR DE "Employee Assistance Programs" OR TI(((employee* OR work OR insurance* OR occupational OR job) N1 (medicin* OR health* OR stress)) OR (Employ* N1 Assistance N1 Program*) OR (employee* N1 Based N1 Service*) OR OSH OR (Occupational N1 safety) OR ((occupational OR worker* OR professional) W0 (health* OR fitness))) OR AB(((employee* OR work OR insurance* OR occupational OR job) N1 (medicin* OR health* OR stress)) OR (Employ* N1 Assistance N1 Program*) OR (employee* N1 Based N1 Service*) OR OSH OR (Occupational N1 safety) OR ((occupational OR worker* OR professional) W0 (health* OR fitness))) OR SU(((employee* OR work OR insurance* OR occupational OR job) N1 (medicin* OR health* OR stress)) OR (Employ* N1 Assistance N1 Program*) OR (employee* N1 Based N1 Service*) OR OSH OR (Occupational N1 safety) OR ((occupational OR worker* OR professional) W0 (health* OR fitness)))

S7 TI(((Compan* OR occupation* OR insurance OR OH OR industrial) W1 (Physician* OR doctor* OR practitioner* OR nurs*)) OR ((labor* OR labour* OR employment OR job) N1 (expert* OR official OR officials OR consultant*))) OR AB(((Compan* OR occupation* OR insurance OR OH OR industrial) W1 (Physician* OR doctor* OR practitioner* OR nurs*)) OR ((labor* OR labour* OR employment OR job) N1 (expert* OR official OR officials OR consultant*))) OR SU(((Compan* OR occupation* OR insurance OR OH OR industrial) W1 (Physician* OR doctor* OR practitioner*)) OR ((labor* OR labour* OR employment OR job) N1 (expert* OR official OR officials OR consultant*)))

S8 (TI((functional W1 (abilit* OR capacit*)) OR (disabilit* N0 manag*)) OR AB((functional W1 (abilit* OR capacit*)) OR (disabilit* N0 manag*)) OR SU((functional W0 (abilit* OR capacit*)) OR (disabilit* N0 manag*))) AND (DE "Employment" OR TI(work OR worker* OR job* OR labor* OR labour* OR occupation* OR vocation* OR employ* OR personnel))

S9 DE "Reentry Workers" OR TI(((return* OR back) N1 to N1 (work OR job* OR labor* OR labour* OR occupation* OR vocation* OR employment)) OR ((work OR worker* OR job* OR labor* OR labour* OR vocation* OR employment OR personnel) N1 (resum* OR reintegrat* OR reentr* OR "re-entry" OR retention OR participat* OR rehab*)) OR (occupation* N0 (resum* OR reintegrat* OR reentr* OR "re-entry" OR retention OR participat* OR rehab*)) OR (stay* N1 at N1 (work OR job* OR labor* OR labour* OR occupation* OR vocation* OR employment)) OR employabilit* OR employable) OR AB(((return* OR back) N1 to N1 (work OR job* OR labor* OR labour* OR occupation* OR vocation* OR employment)) OR ((work OR worker* OR job* OR labor* OR labour* OR vocation* OR employment OR personnel) N1 (resum* OR reintegrat* OR reentr* OR "re-entry" OR retention OR participat* OR rehab*)) OR (occupation* N0 (resum* OR reintegrat* OR reentr* OR "re-entry" OR retention OR participat* OR rehab*)) OR (stay* N1 at N1 (work OR job* OR labor* OR labour* OR occupation* OR vocation* OR employment)) OR employabilit* OR employable) OR SU(((return* OR back) N1 to N1 (work OR job* OR labor* OR labour* OR occupation* OR vocation* OR employment)) OR ((work OR worker* OR job* OR labor* OR labour* OR vocation* OR employment OR personnel) N1 (resum* OR reintegrat* OR reentr* OR "re-entry" OR retention OR participat* OR rehab*)) OR (occupation* N0 (resum* OR reintegrat* OR reentr* OR "re-entry" OR retention OR participat* OR rehab*)) OR (stay* N1 at N1 (work OR job* OR labor* OR labour* OR occupation* OR vocation* OR employment)) OR employabilit* OR employable)

S10 DE "Leaves of Absence" OR DE "Employee Absenteeism" OR DE "Job Performance" OR TI(((sick* OR disability* OR medical) N1 (leave* OR absen*)) OR ((sick OR illness) N1 day*) OR Absenteeism OR presenteeism OR presenteism OR (sickness NEAR/1 presence) OR (working N1 while N1 (sick OR ill)) OR ((work OR worker* OR job* OR labor* OR labour* OR occupation* OR vocation* OR employ* OR personnel) N1 (absen* OR disab* OR disease* OR illness OR impair* OR productivit* OR perform* OR particip* OR abilit* OR loss)) OR Presenteeism OR underemploy* OR (under N1 employ*)) OR AB(((sick* OR disability* OR medical) N1 (leave* OR absen*)) OR ((sick OR illness) N1 day*) OR Absenteeism OR presenteeism OR presenteism OR (sickness NEAR/1 presence) OR (working N1 while N1 (sick OR ill)) OR ((work OR worker* OR job* OR labor* OR labour* OR occupation* OR vocation* OR employ* OR personnel) N1 (absen* OR disab* OR disease* OR illness OR impair* OR productivit* OR perform* OR particip* OR abilit* OR loss)) OR Presenteeism OR underemploy* OR (under N1 employ*)) OR SU(((sick* OR disability* OR medical) N1 (leave* OR absen*)) OR ((sick OR illness) N1 day*) OR Absenteeism OR presenteeism OR presenteism OR (sickness NEAR/1 presence) OR (working N1 while N1 (sick OR ill)) OR ((work OR worker* OR job* OR labor* OR labour* OR occupation* OR vocation* OR employ* OR personnel) N1 (absen* OR disab* OR disease* OR illness OR impair* OR productivit* OR perform* OR particip* OR abilit* OR loss)) OR Presenteeism OR underemploy* OR (under N1 employ*))

S11 S9 OR S10

S12 DE "Health Personnel" OR DE "Allied Health Personnel" OR DE "Emergency Medical Technicians" OR DE "Home Health Aides" OR DE "Mental Health Workers" OR DE "School Psychologists" OR DE "Nurses" OR DE "School Nurses" OR DE "Physicians" OR DE "Foreign Medical Graduates" OR DE "Psychologists" OR DE "School Psychologists" OR TI(health* N1 (professional* OR worker OR workers OR staff OR labourer* OR laborer* OR occupation*) OR Physician* OR doctor* OR practitioner* OR nurs*) OR SU(health* N1 (professional* OR worker OR workers OR staff OR labourer* OR laborer* OR occupation* OR employee*) OR Physician* OR doctor* OR practitioner* OR nurs*)

S13 S11 AND S12

S14 S6 OR S7 OR S8 OR S13

S15 S5 AND S14

GOOGLE SCHOLAR

((interprofessional OR interdisciplinary OR multidisciplinar* OR crossdisciplinar* OR intraprofessional* OR multiprofessional* OR transdisciplin*) AROUND(1) (education OR training)) AND ((Compan* OR occupation* OR insurance OR OH OR industrial) AROUND(1) (Physician* OR doctor* OR practitioner* OR nurs*))

PSYCINFO

S1 MH "Education, Interdisciplinary" OR TI(((Cross OR collaborative OR cooperat* OR "co operat*" OR interprofessional* OR interdisciplinar* OR multidisciplinar* OR crossdisciplinar* OR crossprofession* OR intraprofessional* OR multiprofessional* OR transdisciplin*) N1 (training* OR education*)) OR (joint N1 vocation* N1 train* N1 program*) OR ((inter OR intra OR multi OR trans OR cross ) N1 (professional* OR disciplinar*) NEAR/1 (training* OR education*))) OR AB(((Cross OR collaborative OR cooperat* OR "co operat*" OR interprofessional* OR interdisciplinar* OR multidisciplinar* OR crossdisciplinar* OR crossprofession* OR intraprofessional* OR multiprofessional* OR transdisciplin*) N1 (training* OR education*)) OR (joint N1 vocation* N1 train* N1 program*) OR ((inter OR intra OR multi OR trans OR cross ) NEAR/1 (professional* OR disciplinar*) NEAR/1 (training* OR education*))) OR SU(((Cross OR collaborative OR cooperat* OR "co operat*" OR interprofessional* OR interdisciplinar* OR multidisciplinar* OR crossdisciplinar* OR crossprofession* OR intraprofessional* OR multiprofessional* OR transdisciplin*) N1 (training* OR education*)) OR (joint N1 vocation* N1 train* N1 program*) OR ((inter OR intra OR multi OR trans OR cross ) NEAR/1 (professional* OR disciplinar*) NEAR/1 (training* OR education*)))

S2 DE "Interdisciplinary Treatment Approach" OR TI(interprofessional* OR interdisciplinar* OR multidisciplinar* OR crossdisciplinar* OR crossprofession* OR intraprofessional* OR multiprofessional* OR ((inter OR intra OR multi OR trans OR cross) NEAR/1 (professional* OR disciplinar*)) OR transdisciplin* OR (collaborative NEAR/1 (care OR healthcare) NEAR/1 team*)) OR AB(interprofessional* OR interdisciplinar* OR multidisciplinar* OR crossdisciplinar* OR crossprofession* OR intraprofessional* OR multiprofessional* OR ((inter OR intra OR multi OR trans OR cross) NEAR/1 (professional* OR disciplinar*)) OR transdisciplin* OR (collaborative NEAR/1 (care OR healthcare) NEAR/1 team*)) OR SU(interprofessional* OR interdisciplinar* OR multidisciplinar* OR crossdisciplinar* OR crossprofession* OR intraprofessional* OR multiprofessional* OR ((inter OR intra OR multi OR trans OR cross) NEAR/1 (professional* OR disciplinar*)) OR transdisciplin* OR (collaborative NEAR/1 (care OR healthcare) NEAR/1 team*))

S3 DE "Education" OR DE "Adult Education" OR DE "Continuing Education" OR DE "Personnel Training" OR DE "Inservice Training" OR DE "On the Job Training" OR DE "Training" OR DE "Personnel Training" OR TI(educat* OR cours* OR training OR teach*)

S4 S2 AND S3

S5 S1 OR S4

S6 DE "Occupational Health" OR DE "Work Related Illnesses" OR DE "Occupational Stress" OR TI(((employee* OR work OR insurance* OR occupational OR job) N1 (medicin* OR health* OR stress)) OR (Employ* N1 Assistance N1 Program*) OR (employee* N1 Based N1 Service*) OR OSH OR (Occupational N1 safety) OR ((occupational OR worker* OR professional) W0 (health* OR fitness))) OR AB(((employee* OR work OR insurance* OR occupational OR job) N1 (medicin* OR health* OR stress)) OR (Employ* N1 Assistance N1 Program*) OR (employee* N1 Based N1 Service*) OR OSH OR (Occupational N1 safety) OR ((occupational OR worker* OR professional) W0 (health* OR fitness))) OR SU(((employee* OR work OR insurance* OR occupational OR job) N1 (medicin* OR health* OR stress)) OR (Employ* N1 Assistance N1 Program*) OR (employee* N1 Based N1 Service*) OR OSH OR (Occupational N1 safety) OR ((occupational OR worker* OR professional) W0 (health* OR fitness)))

S7 TI(((Compan* OR occupation* OR insurance OR OH OR industrial) W1 (Physician* OR doctor* OR practitioner* OR nurs*)) OR ((labor* OR labour* OR employment OR job) N1 (expert* OR official OR officials OR consultant*))) OR AB(((Compan* OR occupation* OR insurance OR OH OR industrial) W1 (Physician* OR doctor* OR practitioner* OR nurs*)) OR ((labor* OR labour* OR employment OR job) N1 (expert* OR official OR officials OR consultant*))) OR SU(((Compan* OR occupation* OR insurance OR OH OR industrial) W1 (Physician* OR doctor* OR practitioner*)) OR ((labor* OR labour* OR employment OR job) N1 (expert* OR official OR officials OR consultant*)))

S8 DE "Reemployment" OR TI(((return* OR back) N1 to N1 (work OR job* OR labor* OR labour* OR occupation* OR vocation* OR employment)) OR ((work OR worker* OR job* OR labor* OR labour* OR vocation* OR employment OR personnel) N1 (resum* OR reintegrat* OR reentr* OR "re-entry" OR retention OR participat* OR rehab*)) OR (occupation* N0 (resum* OR reintegrat* OR reentr* OR "re-entry" OR retention OR participat* OR rehab*)) OR (stay* N1 at N1 (work OR job* OR labor* OR labour* OR occupation* OR vocation* OR employment)) OR employabilit* OR employable) OR AB(((return* OR back) N1 to N1 (work OR job* OR labor* OR labour* OR occupation* OR vocation* OR employment)) OR ((work OR worker* OR job* OR labor* OR labour* OR vocation* OR employment OR personnel) N1 (resum* OR reintegrat* OR reentr* OR "re-entry" OR retention OR participat* OR rehab*)) OR (occupation* N0 (resum* OR reintegrat* OR reentr* OR "re-entry" OR retention OR participat* OR rehab*)) OR (stay* N1 at N1 (work OR job* OR labor* OR labour* OR occupation* OR vocation* OR employment)) OR employabilit* OR employable) OR SU(((return* OR back) N1 to N1 (work OR job* OR labor* OR labour* OR occupation* OR vocation* OR employment)) OR ((work OR worker* OR job* OR labor* OR labour* OR vocation* OR employment OR personnel) N1 (resum* OR reintegrat* OR reentr* OR "re-entry" OR retention OR participat* OR rehab*)) OR (occupation* N0 (resum* OR reintegrat* OR reentr* OR "re-entry" OR retention OR participat* OR rehab*)) OR (stay* N1 at N1 (work OR job* OR labor* OR labour* OR occupation* OR vocation* OR employment)) OR employabilit* OR employable)

S9 DE "Employee Leave Benefits" OR DE "Employee Absenteeism" OR DE "Employee Productivity" OR DE "Job Performance" OR DE "Employee Efficiency" OR DE "Employee Retention" OR TI(((sick* OR disability* OR medical) N1 (leave* OR absen*)) OR ((sick OR illness) N1 day*) OR Absenteeism OR presenteeism OR presenteism OR (sickness NEAR/1 presence) OR (working N1 while N1 (sick OR ill)) OR ((work OR worker* OR job* OR labor* OR labour* OR occupation* OR vocation* OR employ* OR personnel) N1 (absen* OR disab* OR disease* OR illness OR impair* OR productivit* OR perform* OR particip* OR abilit* OR loss)) OR Presenteeism OR underemploy* OR (under N1 employ*)) OR AB(((sick* OR disability* OR medical) N1 (leave* OR absen*)) OR ((sick OR illness) N1 day*) OR Absenteeism OR presenteeism OR presenteism OR (sickness NEAR/1 presence) OR (working N1 while N1 (sick OR ill)) OR ((work OR worker* OR job* OR labor* OR labour* OR occupation* OR vocation* OR employ* OR personnel) N1 (absen* OR disab* OR disease* OR illness OR impair* OR productivit* OR perform* OR particip* OR abilit* OR loss)) OR Presenteeism OR underemploy* OR (under N1 employ*)) OR SU(((sick* OR disability* OR medical) N1 (leave* OR absen*)) OR ((sick OR illness) N1 day*) OR Absenteeism OR presenteeism OR presenteism OR (sickness NEAR/1 presence) OR (working N1 while N1 (sick OR ill)) OR ((work OR worker* OR job* OR labor* OR labour* OR occupation* OR vocation* OR employ* OR personnel) N1 (absen* OR disab* OR disease* OR illness OR impair* OR productivit* OR perform* OR particip* OR abilit* OR loss)) OR Presenteeism OR underemploy* OR (under N1 employ*))

S10 (TI((functional W1 (abilit* OR capacit*)) OR (disabilit* N0 manag*)) OR AB((functional W1 (abilit* OR capacit*)) OR (disabilit* N0 manag*)) OR SU((functional W0 (abilit* OR capacit*)) OR (disabilit* N0 manag*))) AND (MH "Work+" OR TI(work OR worker* OR job* OR labor* OR labour* OR occupation* OR vocation* OR employ* OR personnel))

S11 S6 OR S7 OR S8 OR S9 OR S10

S12 S5 AND S11

PUBMED

#1 "Interprofessional Education"[Mesh] OR “Cross training*”[tiab] OR “Cross education*”[tiab] OR “cooperative educat*”[tiab] OR “collaborative educat*”[tiab] OR “co operative train*”[tiab] OR “co operative educat*”[tiab] OR joint vocational training program*[tiab]

#2 interprofessional*[tiab] or interdisciplinar*[tiab] or multidisciplinary*[tiab] OR intraprofessional*[tiab] OR multiprofessional*[tiab] OR "crossdisciplinar*"[tiab] OR "crossprofession*"[tiab] OR "inter professional*"[tiab] or "inter disciplinar*"[tiab] or "intra disciplinar*"[tiab] or "multi disciplinar*"[tiab] OR "multi professional*"[tiab] OR “intra professional*”[tiab] OR transdisciplin*[tiab] OR "cross disciplinar*"[tiab] OR "cross professional*"[tiab] OR "collaborative care team*"[tiab] OR "collaborative healthcare team*"[tiab]

#3 "Education"[Mesh] OR "education" [Subheading] OR education*[ti] OR cours*[ti] OR training[ti] OR teach*[ti]

#4 #2 AND #3

#5 #1 OR #4

#6 "Occupational Health"[Mesh] OR "Occupational Medicine"[Mesh] OR "Occupational Stress"[Mesh] OR "Occupational safety and health*"[tiab] OR "Employee Assistance Program*"[tiab] OR "employee health*"[tiab] OR "Employee stress*"[tiab] OR "Employees health*"[tiab] OR "Employees stress*"[tiab] OR "Employment-Based Service*"[tiab] OR "Insurance health*"[tiab] OR "Insurance medicin*"[tiab] OR "Job Fitness*"[tiab] OR "Job health*"[tiab] OR "Job stress*"[tiab] OR "Occupational fitness*"[tiab] OR "occupational health*"[tiab] OR "occupational medicin*"[tiab] OR "occupational safety"[tiab] OR "occupational stress*"[tiab] OR "OSH"[tiab] OR "Professional fitness*"[tiab] OR "Professional health*"[tiab] OR "social medicin*"[tiab] OR "Worker fitness*"[tiab] OR "Worker health*"[tiab] OR "Worker stress*"[tiab] OR "Workers fitness*"[tiab] OR "Workers health*"[tiab] OR "Workers medicin*"[tiab] OR "Workers stress*"[tiab] OR "Workforce health*"[tiab] OR "Workforce stress*"[tiab] OR "Workplace fitness*"[tiab] OR "Workplace health*"[tiab] OR "Workplace stress*"[tiab] OR "Worksite fitness*"[tiab] OR "Worksite health*"[tiab] OR "Worksite stress*"[tiab]

#7 "Occupational Health Physicians"[Mesh] OR "Occupational Health Nursing"[Mesh] OR "Occupational Health Services"[Mesh] OR "Company doctor*"[tiab] OR "Company nurs*"[tiab] OR "Company Physician*" [tiab] OR "Company practitioner*"[tiab] OR "employment consultant*"[tiab] OR "employment expert*"[tiab] OR "industrial doctor*"[tiab] OR "industrial nurs*"[tiab] OR "industrial physician*"[tiab] OR "industrial practitioner*"[tiab] OR "insurance doctor*"[tiab] OR "insurance nurs*"[tiab] OR "insurance physician*"[tiab] OR "insurance practitioner*"[tiab] OR "job consultant*"[tiab] OR "labor consultant*"[tiab] OR "labor expert*"[tiab] OR "labor official*"[tiab] OR "labor doctor*"[tiab] OR "labor practitioner*"[tiab] OR "labor physician*"[tiab] OR "labor nurs*"[tiab] OR "labour doctor*"[tiab] OR "labour practitioner*"[tiab] OR "labour physician*"[tiab] OR "labour nurs*"[tiab] OR "labour consultant*"[tiab] OR "labour expert*"[tiab] OR "labour official*"[tiab] OR "occupational consultant*"[tiab] OR "occupational doctor*"[tiab] OR "Occupational health doctor*"[tiab] OR "Occupational health physician*"[tiab] OR "Occupational health practitioner*"[tiab] OR "Occupational health nurs*"[tiab] OR "Occupational health service*"[tiab] OR "Occupational healthcare doctor*"[tiab] OR "Occupational healthcare nurs*"[tiab] OR "Occupational healthcare physician*"[tiab] OR "Occupational healthcare practitioner*"[tiab] OR "Occupational healthcare service*"[tiab] OR "occupational nurse*"[tiab] OR "Occupational physician*"[tiab] OR "occupational practitioner*"[tiab] OR "OH doctor*"[tiab] OR "OH nurs*"[tiab] OR "OH physician*"[tiab] OR "OH practitioner*"[tiab] OR "OH service*"[tiab] OR "workplace doctor*"[tiab] OR "workplace nurs*"[tiab] OR "workplace physician*"[tiab] OR "workplace practitioner*"[tiab] OR "worksite doctor*"[tiab] OR "worksite nurs*"[tiab] OR "worksite physician*"[tiab] OR "worksite practitioner*"[tiab] OR "jobsite doctor*"[tiab] OR "jobsite nurs*"[tiab] OR "jobsite physician*"[tiab] OR "jobsite practitioner*"[tiab]

#8 "Return to Work"[Mesh] OR "Rehabilitation, Vocational"[Mesh] OR "back to work*"[tiab] OR "return to the workplace"[tiab] OR "return to work*"[tiab] OR "returning to work*"[tiab] OR "back to work*"[tiab] OR "Job Re-Entry"[tiab] OR "Job ReEntry"[tiab] OR "job reintegrat*"[tiab] OR "job retention*"[tiab] OR "resumption of work*"[tiab] OR "returned to work*"[tiab] OR "work reintegrat*"[tiab] OR employabilit*[tiab] OR employable[tiab] OR "stay at work*"[tiab] OR "vocational rehabilitat*"[tiab] OR "work participat*"[tiab] OR "work reentry"[tiab] OR "work re-entry"[tiab] OR "work resum*"[tiab] OR "work retention"[tiab] OR (return[ti] AND (work*[ti] OR job[ti] OR jobs[ti] OR occupation* OR vocation*[ti]OR labor*[ti] OR labour*[ti] OR employment*[ti]))

#9 "Sick Leave"[Mesh] OR "Absenteeism"[Mesh] OR "Presenteeism"[Mesh] OR "Work Performance"[Mesh] OR "Occupational Diseases"[Mesh:NoExp] OR "disability leav*"[tiab] OR "Illness day*"[TIAB] OR "Job absen*"[TIAB] OR "Job loss"[TIAB] OR "job disabilit*"[TIAB] OR "job illness*" [tiab] OR "job impair*" [tiab] OR "Occupational absen"[TIAB] OR "Occupational disease"[TIAB] OR "Occupational disab"[TIAB] OR "Occupational illness"[TIAB] OR "Occupational impair*"[TIAB] OR "Occupational loss"[TIAB] OR "Occupational sick"[TIAB] OR "medical absen*"[TIAB] OR "medical leave"[TIAB] OR "sick day*"[tiab] OR "sickness day*"[tiab] OR "sickness leave*"[tiab] OR "sick leave*"[tiab] OR "Sickness absen*"[TIAB] OR "under employ*"[tiab] OR "Vocational absen"[TIAB] OR "Vocational disease"[TIAB] OR "Vocational disab"[TIAB] OR "Vocational illness"[TIAB] OR "Vocational impair*"[TIAB] OR "Vocational loss"[TIAB] OR "Vocational sick"[TIAB] OR "work abilit*"[TIAB] OR "Work absen*"[TIAB] OR "Work disabilit*"[TIAB] OR "work functioning"[tiab] OR "work illness*" [tiab] OR "work impair*" [tiab] OR "Work loss"[TIAB] OR "work participation"[tiab] OR "work performance*"[tiab] OR "work related diseas*"[tiab] OR "workplace health*"[tiab] OR "workplace productivit*"[tiab] OR Absenteeism[TIAB] OR Presenteeism[tiab] OR Presenteism[tiab] OR underemploy*[tiab]

#10 (reintegrat*[tiab] OR functional abilit*[tiab] OR functional capacit*[tiab] OR disability manag*[tiab]) AND ("Work"[Mesh] OR work*[ti] OR labor*[ti] OR labour*[ti] OR occupation*[ti] OR employee*[ti] OR personell*[ti])

#11 #6 OR #7 OR #8 OR #9 OR #10

#12 #5 AND #11

WEB OF SCIENCE

1 TS=(((Cross OR collaborative OR cooperat* OR "co operat*" OR interprofessional* OR interdisciplinar* OR multidisciplinar* OR crossdisciplinar* OR crossprofession* OR intraprofessional* OR multiprofessional* OR transdisciplin*) NEAR/1 (training* OR education*)) OR (joint NEAR/1 vocation* NEAR/1 train* NEAR/1 program*))

2 TS=((inter OR intra OR multi OR trans OR cross ) NEAR/1 (professional* OR disciplinar*) NEAR/1 (training* OR education*))

3 TS=(interprofessional* OR interdisciplinar* OR multidisciplinar* OR crossdisciplinar* OR crossprofession* OR intraprofessional* OR multiprofessional* OR ((inter OR intra OR multi OR trans OR cross) NEAR/1 (professional* OR disciplinar*)) OR transdisciplin* OR (collaborative NEAR/1 (care OR healthcare) NEAR/1 team*))

4 TI=(educat* OR cours* OR training OR teach*)

5 #3 AND #4

6 #1 OR #2 OR #5

7 TS=(((employee* OR work OR insurance* OR occupational OR job) NEAR/1 (medicin* OR health* OR stress)) OR (Employ* NEAR/1 Assistance NEAR/1 Program*) OR (employee* NEAR/1 Based NEAR/1 Service*) OR OSH OR (Occupational NEAR/1 safety) OR "occupational health*" OR "occupational fitness*" OR "worker health*" OR "worker fitness" OR "professional health*")

#8 TS=(((Compan* OR occupation* OR insurance OR OH OR industrial) NEAR/1 (Physician* OR doctor* OR practitioner* OR nurs*)) OR ((labor* OR labour* OR employment OR job) NEAR/1 (expert* OR official OR officials OR consultant*)))

#9 TS=((functional NEXT/1 (abilit* OR capacit*)) OR (disabilit* NEAR/0 manag*)) AND TI=(work OR worker* OR job* OR labor* OR labour* OR occupation* OR vocation* OR employ* OR personnel)

#10 TS=(((return* OR back) NEAR/1 to NEAR/1 (work OR job* OR labor* OR labour* OR occupation* OR vocation* OR employment)) OR ((work OR worker* OR job* OR labor* OR labour* OR vocation* OR employment OR personnel) NEAR/1 (resum* OR reintegrat* OR reentr* OR "re-entry" OR retention OR participat* OR rehab*)) OR (occupation* NEXT/0 (resum* OR reintegrat* OR reentr* OR "re-entry" OR retention OR participat* OR rehab*)) OR (stay* NEAR/1 at NEAR/1 (work OR job* OR labor* OR labour* OR occupation* OR vocation* OR employment)) OR employabilit* OR employable)

#11 TS=(((sick* OR disability* OR medical) NEAR/1 (leave* OR absen*)) OR ((sick OR illness) NEAR/1 day*) OR Absenteeism OR presenteeism OR presenteism OR (sickness NEAR/1 presence) OR (working NEAR/1 while NEAR/1 (sick OR ill)) OR ((work OR worker* OR job* OR labor* OR labour* OR occupation* OR vocation* OR employ* OR personnel) NEAR/1 (absen* OR disab* OR disease* OR illness OR impair* OR productivit* OR perform* OR particip* OR abilit* OR loss)) OR Presenteeism OR underemploy* OR (under NEAR/1 employ*))

#12 #10 OR #11

#13 TS=(health* NEAR/1 (professional* OR worker OR workers OR staff OR labourer* OR laborer* OR occupation* OR employee*) OR Physician* OR doctor* OR practitioner* OR nurs*)

#14 #12 AND #13

#15 #7 OR #8 OR #9 OR #14

#16 #6 AND #15
